# Dried fruit intake causally protects against low back pain: A Mendelian randomization study

**DOI:** 10.3389/fnut.2023.1027481

**Published:** 2023-03-23

**Authors:** Jian Huang, Zheng-Fu Xie

**Affiliations:** ^1^Clinical Laboratory Center, The First Affiliated Hospital of Guangxi Medical University, Nanning, China; ^2^Geriatrics Department, The First Affiliated Hospital of Guangxi Medical University, Nanning, China

**Keywords:** Mendelian randomization, low back pain, dried fruit intake, genome-wide association studies, summary statistics

## Abstract

**Background:**

Low back pain is the leading cause of years lived with disability worldwide. The aim of this study was to evaluate whether dried fruit intake causally protects against low back pain using two-sample Mendelian randomization (MR).

**Methods:**

We obtained summary-level data for dried fruit intake (*N* = 421,764) from the IEU Open GWAS Project. Forty-one independent genetic variants proxied dried fruit intake. The corresponding data for low back pain were derived from the FinnGen project (13,178 cases and 164,682 controls; discovery data) and the Neale lab (5,423 cases and 355,771 controls; replication data). We conducted univariable and multivariable MR analyses.

**Results:**

In the univariable MR analysis, the inverse variance weighted estimate showed that greater dried fruit intake was associated with decreased risk of low back pain [odds ratio (OR) = 0.435, 95% confidence interval (CI): 0.287–0.659, *P* = 8.657 × 10^−5^]. Sensitivity analyses using the MR-Egger (OR = 0.078, 95% CI: 0.013–0.479, *P* = 0.009), maximum likelihood (OR = 0.433, 95% CI: 0.295–0.635, *P* = 1.801 × 10^−5^), weighted median (OR = 0.561, 95% CI: 0.325–0.967, *P* = 0.038) and Mendelian Randomization Pleiotropy RESidual Sum and Outlier (MR-PRESSO) (OR = 0.454, 95% CI: 0.302–0.683, *P* = 4.535 × 10^−4^) methods showed consistent results. No evidence of directional pleiotropy was identified according to the Egger intercept (intercept *P*-value = 0.065) or applying the MR-PRESSO method (global test *P*-value = 0.164). The replication analysis yielded similar results. The multivariable MR revealed that the inverse association between dried fruit intake and low back pain was consistent after adjustment for fresh fruit intake, body mass index, current tobacco smoking, alcohol intake frequency, total body bone mineral density, serum 25-hydroxyvitamin D levels, and vigorous physical activity.

**Conclusion:**

This MR study provides evidence to support that dried fruit intake causally protects against low back pain.

## Introduction

Low back pain is a leading contributor to disability worldwide among all musculoskeletal disorders (1). For the majority of patients, low back pain is non-specific, because precise identification of the specific nociceptive source is not possible. If lacking proper diagnosis and therapy, acute low back pain cases are at risk for the development of chronic pain. This can lead to more frequent healthcare visits, increased financial costs, activity limitation, high rates of disability, and reduced quality of life. The pathogenesis of low back pain is complex and multifactorial. Numerous risk factors contribute to its pathogenesis, including an older age, unhealthy lifestyles, physical factors, musculoskeletal tissue structural failure, and psychological factors (1). In addition, low back pain can be associated with some diseases such as tumors and infections. Epidemiological studies show that low back pain affects about 80% of people in Western countries during their lifetime (2). In Europe, the prevalence of low back pain has ranged between 6 and 11% in the general population (3). The social and economic costs related to low back pain are enormous. It is estimated that the annual economic loss caused by low back pain is about 2.8 billion pounds in the United Kingdom and 8.9 billion euros in Spain (1, 4). Low back pain has become a significant burden for society and health systems, and this burden is still rising due to an aging population.

Dried fruits are shelf-stable forms of fresh fruits, which contain low water content. They represent a small but significant proportion of human diets in modern populations. Traditional dried fruits include prunes, pears, peaches, apples, dates, raisins, mulberries, figs, and apricots. The Middle East and North Africa region has the highest per capita dried fruit consumption (>30 kg per year) (5). In contrast, dried fruit consumption is low in Europe (5). For instance, per capita consumption of dried grapes is about 1.08 kg per year in Germany.^1^ Dried fruits are enriched in a variety of dietary fibers, and they are good sources of a number of micronutrients, including magnesium, potassium, iron, calcium, phosphorus, zinc, niacin, vitamin K, vitamin B6, vitamin E, and choline (6). In addition, dried fruits contain many bioactive compounds, including polyphenols, carotenoids, and flavonoids (6). Over the two past decades, experimental research and human clinical studies have reported beneficial effects of dried fruit intake on reducing inflammation, body weight, blood pressure, and glycated hemoglobin levels (7–11). In addition, dried fruits have been shown to exert anti-oxidative, anti-cancer, and anti-aging properties (5, 12–15). However, it remains unclear whether dried fruit intake has a beneficial effect on low back pain. Mendelian randomization (MR) is a statistical method using single nucleotide polymorphisms (SNPs) as instruments for inferring causality between risk factors and a disease outcome. MR can greatly reduce the risk of confounding and reverse causation, which are shortcomings of conventional epidemiological studies (16). Recently, Jin et al. (17) used inverse variance weighted MR and weighted median methods to evaluate the causality between dried fruit intake and 11 site-specific cancers, finding that dried fruit intake had protective effects against some site-specific cancers such as breast cancer and lung cancer. In the present study, we aimed to analyze the potential causal effect of genetically predicted dried fruit intake on low back pain applying the MR framework.

## Methods

### Ethical approval

Our study was exempt from ethical approval, because we only analyzed publicly available summary-level data of genome-wide association studies (GWAS). All GWAS summary statistics used in our MR study were obtained from the IEU Open GWAS Project^2^ (18).

### Genetic instruments

Summary-level data for dried fruit intake were obtained from a GWAS study (*N* = 421,764) of the UK Biobank, using the GWAS-ID “ukb-b-16576.” The UK Biobank is a large population-based cohort of more than 500,000 participants who were recruited at ages 40 to 69 years across England, Wales, and Scotland from 2006 to 2010 (19). In the UK Biobank, dried fruit intake was available from a question “About how many pieces of dried fruit would you eat per day? (Count one prune, one dried apricot, 10 raisins as one piece; put 0 if you do not eat any)” included in the touchscreen questionnaire^3^ Participants who answered >100 were rejected. Genotyping of the participants was performed using Affymetrix UK Biobank Axiom array. Extensive centralized quality control was applied for the genetic data (19). We identified SNPs robustly associated with dried fruit intake at genome-wide significance (*P* < 5 × 10^−8^) as instrumental variables. We restricted instrumental variables to independent SNPs without linkage disequilibrium (*R*^2^ < 0.001) to minimize MR biases by using the clump_data function of the R package “TwoSampleMR” version 0.5.6^4^ (18, 20). The European panel of 1,000 Genomes data was used as the reference panel (21). Palindromic SNPs with intermediate allele frequencies were not used, because they may invert the direction of causality.

Analyses were adjusted for fresh fruit intake, body mass index (BMI), current smoking status, alcohol intake frequency, total body bone mineral density, serum 25-hydroxyvitamin D levels, and vigorous physical activity applying multivariable MR. Summary-level data for these exposures were obtained from the IEU Open GWAS Project. The detailed information are shown in Table 1.

**Table 1:** Detailed information on the GWAS datasets used in this MR study.

**Dataset type**	**Item**	**GWAS ID**	**Author**	**Consortium**	**Year**	**Population**	**Sample size**	**Sex**
Exposure	Dried fruit intake	ukb-b-16576	Ben Elsworth	MRC-IEU	2018	European	421,764	Males and females
	Fresh fruit intake	ukb-b-3881	Ben Elsworth	MRC-IEU	2018	European	446,462	Males and females
	Body mass index	ieu-b-40	Yengo L	GIANT	2018	European	681,275	Males and females
	Current tobacco smoking	ukb-b-223	Ben Elsworth	MRC-IEU	2018	European	462,434	Males and females
	Alcohol intake frequency	ukb-b-5779	Ben Elsworth	MRC-IEU	2018	European	462,346	Males and females
	Total body bone mineral density	ebi-a-GCST005348	Medina-Gomez C	NA	2018	European	56,284	Males and females
	Serum 25-hydroxyvitamin D levels	ebi-a-GCST90000618	Revez JA	NA	2020	European	496,946	Males and females
	Vigorous physical activity	ebi-a-GCST006098	Klimentidis YC	NA	2018	European	261,055	NA
Outcome	Low back pain (discovery)	finn-b-M13_LOWBACKPAIN	NA	FinnGen	2021	European	13,178 cases and 164,682 controls	Males and females
	Low back pain (replication)	ukb-d-M13_LOWBACKPAIN	Neale lab	NA	2018	European	5,423 cases and 355,771 controls	Males and females

MRC-IEU, Medical Research Center-Integrative Epidemiology Unit; SNP, single nucleotide polymorphism; GWAS, genome wide association study; MR, mendelian randomization; GIANT, Genetic Investigation of ANthropometric Traits; NA, not applicable.

### Summary-level data for low back pain

Summary-level data for low back pain in individuals of European descent were derived from the FinnGen study (13,178 cases and 164,682 controls) using the GWAS-ID “finn-b-M13_LOWBACKPAIN” (Table 1). The FinnGen study is a nationwide cohort launched in 2017. It aims to collect and evaluate genome and health data from 500,000 Finnish biobank participants (22). Low back pain was identified according to International classification of diseases (ICD) codes retrieved from nationwide registries in Finland. The effect alleles in dried fruit intake and low back pain datasets were harmonized using the harmonize_data function from the TwoSampleMR R package.

For replication analyses, summary statistics for low back pain were obtained from the Neale lab study with the GWAS-ID “ukb-d-M13_LOWBACKPAIN”, including 5,423 cases and 355,771 controls (Table 1).

### Statistical analysis

In this two-sample MR analysis, we used the inverse variance weighted method implemented in the TwoSampleMR R package as the primary MR method. This method gives reliable causal assessments and has the highest statistical power if the selected SNPs meet the instrumental variable assumptions (23). We then conducted sensitivity analyses using the MR-Egger, weighted median, weighted mode, simple mode, and maximum likelihood methods for assessing the robustness of the findings. These methods relax different MR assumptions regarding pleiotropy. For instance, the MR-Egger method can give unbiased assessments even when the exclusion restriction assumption is violated, but it has comparatively low statistical power (24). The weighted median method stipulates that at least 50% of the information is from valid instrumental variables (25). To assess the presence of horizontal pleiotropy, we used the MR-Egger regression intercept (24). If the intercept term is significantly different from zero, this is taken as evidence for horizontal pleiotropy (24). We applied the Mendelian Randomization Pleiotropy RESidual Sum and Outlier (MR-PRESSO) method to detect and correct for potentially pleiotropic outliers (26). We carried out leave-one-out sensitivity analysis to evaluate whether individual instrumental variables drive observed causal associations. A PhenoScanner^5^ search was performed to identify phenotypes related to the selected instrumental variables. For evaluating the presence of heterogeneity between variant-specific estimates, we used the Cochran's Q statistical test. Besides univariable MR, we performed multivariable MR to control for potential confounders including fresh fruit intake, BMI, current smoking status, alcohol intake frequency, total body bone mineral density, serum 25-hydroxyvitamin D levels, and vigorous physical activity. Seven models for multivariable MR were taken into account: (a) model 1: adjustment for fresh fruit intake; (b) model 2: adjustment for BMI; (c) model 3: adjustment for current tobacco smoking; (d) model 4: adjustment for alcohol intake frequency; (e) model 5: adjustment for total body bone mineral density; (f) model 6: adjustment for serum 25-hydroxyvitamin D levels; and (g) model 7: adjustment for vigorous physical activity. We carried out all MR analyses using the TwoSampleMR (version 0.5.6) and MR-PRESSO (version 1.0) packages in R version 4.0.4. Statistical significance was set at *P* < 0.05.

## Results

Forty-one independent SNPs were selected as instrumental variables in the assessment of dried fruit intake (Supplementary Table S1). These instrument SNPs explained 0.63% of the variance in dried fruit intake. According to the study by Jin et al. (17), the *F*-statistics of individual SNPs ranged between 17.5 and 47.9, indicating adequate instrument strength.

**Figure 1:**
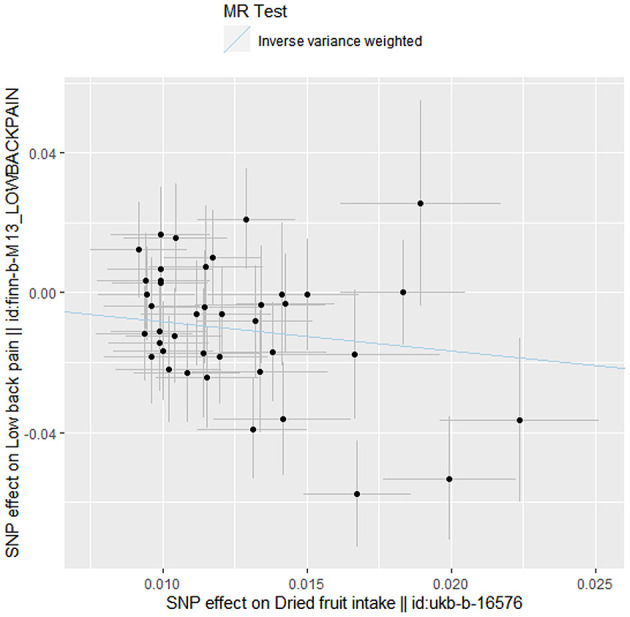
Scatter plot showing Mendelian randomization effect estimates of dried fruit intake over low back pain. Each variant-low back pain association is plotted against variant-dried fruit intake association, and the corresponding Mendelian randomization estimate for inverse variance weighted is plotted.

The inverse variance weighted MR estimate showed that greater dried fruit intake was significantly associated with decreased risk of low back pain (OR = 0.435, 95% CI: 0.287–0.659, *P* = 8.657 × 10^−5^) (Table 2 and Figure 1). Sensitivity analyses using the MR-Egger (OR = 0.078, 95% CI: 0.013–0.479, *P* = 0.009), maximum likelihood (OR = 0.433, 95% CI: 0.295–0.635, *P* = 1.801 × 10^−5^), weighted median (OR = 0.561, 95% CI: 0.325–0.967, *P* = 0.038) and MR-PRESSO (OR = 0.454, 95% CI: 0.302–0.683, *P* = 4.535 × 10^−4^) methods also revealed an inverse association between greater dried fruit intake and low back pain (Table 2). The Cochran's Q statistical test did not provide evidence for statistically significant heterogeneity in the causal estimate amongst instrumental variables (Q = 48.801, *P* = 0.160). No evidence of directional pleiotropy was identified according to the Egger intercept (intercept *P*-value = 0.065) or applying the MR-PRESSO method (global test *P*-value = 0.164). Results of the leave-one-out sensitivity analysis demonstrated that the observed causal associations were not driven by individual instrumental variables (Table 3). We further conducted a PhenoScanner search to identify SNPs related to other potential confounders at genome-wide significance (*P* < 5 × 10^−8^) (Supplementary Table S2). The result of the inverse variance weighted analysis was not significantly altered after removing these SNPs (OR = 0.567, 95% CI: 0.339–0.950, *P* = 0.031).

**Table 2:** MR analysis for the association between dried fruit intake and low back pain.

**Dataset for low back pain**	**Cases/controls**	**Number of instruments**	**MR method**	**OR (95% CI)**	***P*-value**
finn-b-M13_LOWBACKPAIN (Discovery)	13,178/164,682	41	IVW	0.435 (0.287–0.659)	8.657 × 10^−5^
		41	MR-Egger	0.078 (0.013–0.479)	0.009
		41	Maximum likelihood	0.433 (0.295–0.635)	1.801 × 10^−5^
		41	Weighted median	0.561 (0.325–0.967)	0.038
		41	MR-PRESSO	0.454 (0.302–0.683)	4.535 × 10^−4^
ukb-d-M13_LOWBACKPAIN (Replication)	5,423/355,771	41	IVW	0.984 (0.976–0.992)	5.704 × 10^−5^
		41	MR-Egger	0.961 (0.926–0.996)	0.004
		41	Maximum likelihood	0.984 (0.976–0.992)	8.193 × 10^−5^
		41	Weighted median	0.985 (0.974–0.996)	0.009
		41	MR-PRESSO	0.984 (0.977–0.991)	1.240 × 10^−4^

IVW, inverse variance weighted; CI, confidence interval; OR, odds ratio; MR, Mendelian randomization.

**Table 3:** Leave-one-out analysis using the inverse variance weighted method.

	**SNP**	**OR**	**95% lower confidence interval**	**95% upper confidence interval**
All	0.435	0.287	0.659
Removing	rs10026792	0.447	0.293	0.682
Removing	rs10129747	0.438	0.286	0.670
Removing	rs10740991	0.491	0.332	0.726
Removing	rs10896126	0.423	0.277	0.648
Removing	rs11152349	0.424	0.278	0.645
Removing	rs11586016	0.440	0.288	0.672
Removing	rs11632215	0.429	0.281	0.655
Removing	rs11720884	0.432	0.282	0.661
Removing	rs11772627	0.414	0.270	0.636
Removing	rs11811826	0.432	0.282	0.662
Removing	rs12137234	0.445	0.292	0.678
Removing	rs1582322	0.426	0.279	0.650
Removing	rs1622515	0.425	0.279	0.649
Removing	rs1648404	0.426	0.279	0.649
Removing	rs17175518	0.424	0.279	0.646
Removing	rs17184707	0.431	0.282	0.659
Removing	rs1797235	0.442	0.289	0.675
Removing	rs2328887	0.420	0.277	0.637
Removing	rs2533273	0.437	0.286	0.669
Removing	rs261809	0.444	0.291	0.677
Removing	rs3101339	0.425	0.277	0.651
Removing	rs34162196	0.447	0.292	0.684
Removing	rs3764002	0.466	0.309	0.702
Removing	rs4140799	0.428	0.280	0.654
Removing	rs4149513	0.414	0.273	0.629
Removing	rs4269101	0.441	0.287	0.676
Removing	rs429358	0.476	0.316	0.718
Removing	rs4800488	0.444	0.290	0.680
Removing	rs57499472	0.413	0.274	0.623
Removing	rs62084586	0.429	0.280	0.656
Removing	rs72720396	0.439	0.287	0.671
Removing	rs746868	0.403	0.269	0.604
Removing	rs75641275	0.457	0.301	0.694
Removing	rs7582086	0.431	0.282	0.659
Removing	rs7599488	0.438	0.286	0.671
Removing	rs7808471	0.448	0.294	0.684
Removing	rs7829800	0.418	0.276	0.632
Removing	rs8081370	0.438	0.286	0.671
Removing	rs862227	0.419	0.277	0.635
Removing	rs893856	0.443	0.290	0.677
Removing	rs9385269	0.430	0.281	0.660

SNP, single nucleotide polymorphism; OR, odds ratio.

We replicated the protective effect of dried fruit intake on low back pain using a validation sample from the Neale lab study (GWAS ID: ukb-d-M13_LOWBACKPAIN). Consistent with the primary analysis, the replication analysis showed that greater dried fruit intake was causally associated with decreased low back pain risk (Table 2). Besides dried fruit intake, we evaluated the casual association of fresh fruit intake, BMI, current tobacco smoking, alcohol intake frequency, total body bone mineral density, serum 25-hydroxyvitamin D levels, and vigorous physical activity with low back pain using the inverse variance weighted method (Supplementary Table S3). The instrumental variables for these exposures and their association with low back pain are shown in Supplementary Tables S4–S10.

To verify whether the protective effect of dried fruit intake on low back pain was independent of fresh fruit intake, BMI, current tobacco smoking, alcohol intake frequency, total body bone mineral density, serum 25-hydroxyvitamin D levels, and vigorous physical activity, we performed multivariable MR analyses. Results of multivariable MR analyses supported that greater dried fruit intake was protective against low back pain (Table 4).

**Table 4:** Results of multivariable MR.

**Exposure**	**Outcome**	**OR (95% CI)**	***P*-value**	**Model**	**Controlling for**
Dried fruit intake	Low back pain	0.487 (0.292–0.810)	0.006	1	Fresh fruit intake
Dried fruit intake	Low back pain	0.417 (0.274-0.633)	3.937 × 10^−5^	2	BMI
Dried fruit intake	Low back pain	0.486 (0.303–0.779)	0.003	3	Current tobacco smoking
Dried fruit intake	Low back pain	0.504 (0.323–0.788)	0.003	4	Alcohol intake frequency
Dried fruit intake	Low back pain	0.353 (0.210–0.595)	9.144 × 10^−5^	5	Total body bone mineral density
Dried fruit intake	Low back pain	0.470 (0.313–0.705)	2.597 × 10^−4^	6	Serum 25-Hydroxyvitamin D levels
Dried fruit intake	Low back pain	0.483 (0.303–0.771)	0.002	7	Vigorous physical activity

MR, Mendelian randomization; OR, odds ratio; CI, confidence interval; BMI, body mass index.

## Discussion

To our knowledge, this is the first MR study to evaluate the casual association between dried fruit intake and low back pain. Using genetic data from individuals of European descent, our analyses showed that greater dried fruit intake was associated with decreased low back pain risk. This association was consistent after adjustment for fresh fruit intake, BMI, current tobacco smoking, alcohol intake frequency, total body bone mineral density, serum 25-hydroxyvitamin D levels, and vigorous physical activity.

MR is an effective analytic method for causal inference. It is less affected by certain fundamental shortcomings of traditional observational investigations. Recently, MR studies have been performed to assess potential risk factors for low back pain. In 2020, Elgaeva and colleagues applied inverse variance weighted meta-analysis as the main method for evaluating the causal association between BMI and back pain (27). Summary statistics for BMI were obtained from the GIANT consortium (*N* = 322,154), and the corresponding data for back pain and chronic back pain were derived from a large European sample (*N* = 453,860). They found that 1-standard deviation (4.65 kg/m^2^) increase in BMI was associated with a significant increase in back pain (OR = 1.15, 95% CI: 1.06–1.25, *P* = 0.001) and chronic back pain (OR = 1.20, 95% CI: 1.09–1.32, *P* = 0.0002); the significant causal association remained in secondary analysis and sensitivity analyses. These results suggested that a higher BMI may be a risk factor for back pain. Consistent with their findings, Zhou and colleagues found a casual association between BMI and low back pain (OR = 1.28, 95% CI: 1.18–1.39, *P* = 6.60 × 10^−9^) using a two-sample MR design (28). An MR study by a Chinese research group recently evaluated the causal effect of plasma omega-3 levels on low back pain risk, finding that up-regulated plasma omega-3 levels were linked with reduced low back pain risk using the inverse variance weighted method (β = −0.366, OR = 0.694, *P* = 0.049) (29). However, this link was not supported by sensitivity analyses using the weighted mode (*P* = 0.281) and MR-Egger (*P* = 0.228) methods. In 2022, Williams et al. applied inverse weighted variance, Causal Analysis Using Summary Effect (CAUSE), and sensitivity analyses to evaluate risk factors for chronic back pain (30). Their study demonstrated that several life style factors including greater alcohol intake (inverse variance weighted OR = 1.29, 95% CI: 1.17–1.43, *P* = 7.2 × 10^−7^) and smoking (inverse variance weighted OR = 1.27, 95% CI: 1.19–1.35, *P* = 7.0 × 10^−15^) increased the risk of chronic back pain (30).

In our study, we applied a two-sample MR design to evaluate the causal association between dried fruit intake and low back pain. Consistent with previous MR studies, we used the inverse variance weighted method in the primary MR analysis. The result of the inverse variance weighted-based estimate was statistically significant; no single instrument SNPs drove the causal estimate. Multiple sensitivity analyses using various methods revealed consistent and stable results. To verify the causal association found in the discovery dataset, we used a validation sample for low back pain from another European population. The replication analysis provided similar results and suggested a causal association between dried fruit intake and low back pain. Furthermore, to adjust for potential confounding factors including fresh fruit intake, BMI, current tobacco smoking, alcohol intake frequency, total body bone mineral density, serum 25-hydroxyvitamin D levels, and vigorous physical activity, we used multivariable MR. The effect of dried fruit intake remained after adjustment for these factors. In summary, the above-mentioned efforts enhanced the robustness of the results in our study.

The mechanisms involved in the causal association between dried fruit intake and low back pain remain unclear. Dried fruits are obtained from fresh fruits by using various drying techniques. They are important healthful snacks and are rich sources of dietary fibers, minerals, vitamins, and a variety of bioactive compounds such as flavonoids and carotenoids (6). Dried fruits exert multiple biological effects, including anti-oxidative, anti-inflammatory, anti-atherosclerosis, and anti-cancer effects (5, 7, 10, 12, 14, 15, 31). Experimental research showed that dried fruit intake suppressed proinflammatory cytokines and promoted functions of the musculoskeletal system (32–34). In clinical studies, numerous authors found that daily intake of dried fruits had protective effects on musculoskeletal health in both men and women (35–37). The protective effect of dried fruit intake on low back pain might be related to the micronutrients and bioactive compounds dried fruits contain. For instance, vitamin E could improve musculoskeletal health by maintaining bone mineral density and reducing oxidative stress and inflammation (38, 39). In addition, some authors found that flavonoids had antioxidant and antinociceptive activities, which may be used to relieve pain (40, 41). However, since the evidence regarding the underlying mechanisms is limited, further experimental and clinical studies are required.

One limitation of this MR study is that we did not have access to individual-level data to adjust for medication usage. It has been reported that some medications such as non-steroidal anti-inflammatory drugs and opioids have beneficial effects for low back pain. Another limitation is that we only studied participants of European descent in this MR study. The association between dried fruit intake and low back pain in Asians such as Chinese and Indians remains unclear.

In summary, this MR study using genetic data from individuals of European descent provided evidence to support that greater dried fruit intake was associated with decreased risk of low back pain. The results highlighted the importance of evaluating dried fruit intake for the prevention of low back pain. Further validations using randomized controlled trials with large sample sizes are warranted.

## Data availability statement

Publicly available datasets were analyzed in this study. They can be obtained at https://gwas.mrcieu.ac.uk/.

## Ethics statement

Our Mendelian randomization study analyzing publicly available summary-level data was exempt from ethical approval.

## Author contributions

JH contributed to study concept and design, acquisition and interpretation of data, statistical analyses, and manuscript writing and revision. Z-FX supervised the study, contributed to data interpretation, and assisted in reviewing the manuscript. Both authors read and approved the final manuscript.

## Footnotes

^1^ https://gfa.org.ge/wp-content/uploads/2019/05/Dried-Fruits-market-research.pdf

^2^ https://gwas.mrcieu.ac.uk/

^3^ https://biobank.ndph.ox.ac.uk/ukb/field.cgi?id=1319

^4^ https://github.com/MRCIEU/TwoSampleMR

^5^ http://www.phenoscanner.medschl.cam.ac.uk/
