# Supplementary material for: Dried fruit intake causally protects against low back pain: A Mendelian randomization study
Source: Front Nutr. 2023 Mar 23;10:1027481. doi: 10.3389/fnut.2023.1027481 (PMC10076586; doi:10.3389/fnut.2023.1027481)
Supplement: Supplementary file 1 [file Table_1.DOCX]

Supplementary Table S1 Characteristics of the instruments for dried fruit intake and their associations with low back pain.

| **SNP** | **Chr** | **Position** | **EA** | **OA** | **Exposure effect** |  |  |  | **Outcome effect** |  |  |
| --- | --- | --- | --- | --- | --- | --- | --- | --- | --- | --- | --- |
|  |  |  |  |  | **β** | **SE** | ***P*** |  | **β** | **SE** | ***P*** |
| rs10026792 | 4 | 2862190 | A | G | 0.011 | 0.002 | 3.90E-09 |  | -0.023 | 0.014 | 0.103 |
| rs10129747 | 14 | 77433198 | G | A | 0.009 | 0.002 | 2.60E-08 |  | -0.012 | 0.014 | 0.388 |
| rs10740991 | 10 | 22058137 | C | G | 0.017 | 0.002 | 2.00E-19 |  | -0.058 | 0.015 | 0.0002 |
| rs10896126 | 11 | 66292908 | G | A | -0.015 | 0.002 | 1.60E-16 |  | 7.00E-04 | 0.016 | 0.967 |
| rs11152349 | 18 | 60233646 | A | G | 0.010 | 0.002 | 4.90E-08 |  | 0.007 | 0.015 | 0.656 |
| rs11586016 | 1 | 44031793 | C | G | 0.010 | 0.002 | 1.10E-08 |  | -0.014 | 0.014 | 0.300 |
| rs11632215 | 15 | 45327794 | C | A | -0.014 | 0.003 | 4.40E-08 |  | 0.001 | 0.021 | 0.945 |
| rs11720884 | 3 | 43941406 | G | A | 0.011 | 0.002 | 7.60E-09 |  | -0.006 | 0.015 | 0.688 |
| rs11772627 | 7 | 2109821 | C | G | 0.018 | 0.002 | 3.00E-17 |  | 0 | 0.015 | 0.999 |
| rs11811826 | 1 | 204603861 | A | T | 0.013 | 0.002 | 4.40E-11 |  | -0.008 | 0.016 | 0.610 |
| rs12137234 | 1 | 72270797 | T | C | 0.010 | 0.002 | 2.80E-08 |  | -0.022 | 0.015 | 0.145 |
| rs1582322 | 16 | 52105988 | G | A | 0.010 | 0.002 | 6.80E-09 |  | 0.003 | 0.015 | 0.818 |
| rs1622515 | 11 | 95523433 | G | A | 0.010 | 0.002 | 2.90E-09 |  | 0.003 | 0.014 | 0.846 |
| rs1648404 | 4 | 37175523 | T | C | 0.009 | 0.002 | 1.80E-08 |  | 0.003 | 0.014 | 0.808 |
| rs17175518 | 18 | 57850583 | A | C | 0.011 | 0.002 | 5.90E-09 |  | 0.008 | 0.018 | 0.668 |
| rs17184707 | 2 | 166183577 | T | C | -0.011 | 0.002 | 2.10E-08 |  | 0.004 | 0.016 | 0.799 |
| rs1797235 | 15 | 47821612 | C | G | -0.010 | 0.002 | 8.90E-09 |  | 0.017 | 0.014 | 0.239 |
| rs2328887 | 6 | 25430149 | C | T | 0.019 | 0.003 | 8.80E-12 |  | 0.026 | 0.029 | 0.382 |
| rs2533273 | 7 | 153485282 | A | C | -0.010 | 0.002 | 3.90E-09 |  | 0.011 | 0.014 | 0.419 |
| rs261809 | 1 | 241054465 | G | A | -0.010 | 0.002 | 9.80E-09 |  | 0.018 | 0.014 | 0.173 |
| rs3101339 | 1 | 72748669 | C | A | 0.014 | 0.002 | 6.20E-17 |  | -0.003 | 0.014 | 0.817 |
| rs34162196 | 14 | 22038125 | T | C | -0.022 | 0.003 | 7.10E-16 |  | 0.037 | 0.024 | 0.119 |
| rs3764002 | 12 | 108618630 | T | C | 0.013 | 0.002 | 5.10E-12 |  | -0.039 | 0.014 | 0.006 |
| rs4140799 | 14 | 72170969 | A | G | 0.009 | 0.002 | 1.80E-08 |  | -4.00E-04 | 0.014 | 0.979 |
| rs4149513 | 2 | 101022726 | A | G | 0.012 | 0.002 | 2.20E-12 |  | 0.010 | 0.014 | 0.453 |
| rs4269101 | 3 | 18763543 | G | T | -0.014 | 0.002 | 1.10E-13 |  | 0.017 | 0.014 | 0.234 |
| rs429358 | 19 | 45411941 | C | T | 0.020 | 0.002 | 6.70E-18 |  | -0.053 | 0.018 | 0.003 |
| rs4800488 | 18 | 21117571 | A | C | 0.012 | 0.002 | 7.70E-13 |  | -0.019 | 0.014 | 0.171 |
| rs57499472 | 3 | 147239337 | C | T | 0.010 | 0.002 | 8.10E-09 |  | 0.017 | 0.014 | 0.224 |
| rs62084586 | 17 | 56423201 | C | T | 0.013 | 0.002 | 3.20E-09 |  | -0.004 | 0.017 | 0.833 |
| rs72720396 | 1 | 91191582 | G | A | 0.011 | 0.002 | 8.70E-09 |  | -0.017 | 0.018 | 0.346 |
| rs746868 | 6 | 31540429 | G | C | -0.013 | 0.002 | 5.20E-14 |  | -0.021 | 0.014 | 0.141 |
| rs75641275 | 1 | 98327133 | C | A | -0.014 | 0.002 | 2.90E-09 |  | 0.036 | 0.016 | 0.025 |
| rs7582086 | 2 | 60231826 | T | G | -0.010 | 0.002 | 8.80E-09 |  | 0.004 | 0.014 | 0.786 |
| rs7599488 | 2 | 60718347 | T | C | -0.010 | 0.002 | 6.70E-10 |  | 0.012 | 0.014 | 0.365 |
| rs7808471 | 7 | 132716502 | C | T | -0.012 | 0.002 | 1.10E-10 |  | 0.024 | 0.014 | 0.090 |
| rs7829800 | 8 | 144258705 | G | A | -0.010 | 0.002 | 5.10E-09 |  | -0.016 | 0.016 | 0.311 |
| rs8081370 | 17 | 1373612 | T | C | -0.017 | 0.003 | 1.40E-08 |  | 0.018 | 0.019 | 0.341 |
| rs862227 | 16 | 73602926 | G | A | -0.009 | 0.002 | 4.30E-08 |  | -0.012 | 0.014 | 0.372 |
| rs893856 | 10 | 126723567 | A | G | -0.013 | 0.002 | 1.30E-08 |  | 0.023 | 0.017 | 0.193 |
| rs9385269 | 6 | 98547979 | T | C | 0.012 | 0.002 | 7.20E-13 |  | -0.006 | 0.014 | 0.658 |

EA, effect allele; OA, other allele; SNP, single nucleotide polymorphism; SE, standard error.
