# Supplementary material for: Dried fruit intake causally protects against low back pain: A Mendelian randomization study
Source: Front Nutr. 2023 Mar 23;10:1027481. doi: 10.3389/fnut.2023.1027481 (PMC10076586; doi:10.3389/fnut.2023.1027481)
Supplement: Supplementary file 2 [file Table_2.DOCX]

Supplementary Table S2 The SNPs that are related to other potential confounders at genome-wide significance (*P* < 5×10^−8^).

| SNP | Phenotype/Trait |
| --- | --- |
| rs10740991 | Body mass index, waist circumference, hip circumference, etc. |
| rs11720884 | Leg fat percentage left, leg fat percentage right, leg fat mass right, and leg fat mass left |
| rs11772627 | Body mass index, impedance of leg left, overall health rating, etc. |
| rs17175518 | Basal metabolic rate, leg predicted mass left, leg predicted mass right, etc. |
| rs3101339 | Leg fat mass left, body mass index, whole body fat mass, etc. |
| rs3764002 | Body fat percentage, impedance of leg left, trunk fat percentage, etc. |
| rs4149513 | Years of educational attainment, body fat percentage, leg fat percentage left, etc. |
| rs4269101 | Body fat percentage, arm fat percentage right, arm fat percentage left, and trunk fat percentage |
| rs429358 | Late onset Alzheimers disease, dementia with Lewy bodies, self-reported high cholesterol, etc. |
| rs4800488 | Weight, basal metabolic rate, leg fat mass left, etc. |
| rs72720396 | Alcohol usually taken with meals, morning vs evening chronotype, morning or evening person, etc. |
| rs746868 | Treatment with insulin product, IgA deficiency, rheumatoid arthritis, etc. |
| rs75641275 | Schizophrenia, leg fat mass right, waist circumference, etc. |
| rs7599488 | Red blood cell count, F cell distribution, time spent watching television, etc. |
| rs9385269 | Leg fat percentage right, body fat percentage, time spent using computer, etc. |

SNPs, single nucleotide polymorphisms.
