# Supplementary material for: Dried fruit intake causally protects against low back pain: A Mendelian randomization study
Source: Front Nutr. 2023 Mar 23;10:1027481. doi: 10.3389/fnut.2023.1027481 (PMC10076586; doi:10.3389/fnut.2023.1027481)
Supplement: Supplementary file 3 [file Table_3.DOCX]

Supplementary Table S3 Estimating the association between other exposures and low back pain using the IVW method.

| Exposure | Number of SNPs | OR (95% CI) | IVW *P*-value | MR-Egger intercept *P*-value |
| --- | --- | --- | --- | --- |
| Fresh fruit intake | 51 | 0.594 (0.358-0.987) | 0.044 | 0.806 |
| Body mass index | 466 | 1.319 (1.196-1.455) | 3.066×10^-8^ | 0.714 |
| Current tobacco smoking | 34 | 3.908 (1.944-7.855) | 1.301×10^-4^ | 0.891 |
| Alcohol intake frequency | 91 | 1.325 (1.140-1.540) | 2.455×10^-4^ | 0.787 |
| Total body bone mineral density | 80 | 1.002 (0.924-1.087) | 0.964 | 0.924 |
| Serum 25-Hydroxyvitamin D levels | 113 | 1.047 (0.931-1.179) | 0.441 | 0.243 |
| Vigorous physical activity | 7 | 0.521 (0.147-1.844) | 0.312 | 0.810 |

IVW, inverse variance weighted; CI, confidence interval; OR, odds ratio; MR, mendelian randomization.
