# Supplementary material for: Dried fruit intake causally protects against low back pain: A Mendelian randomization study
Source: Front Nutr. 2023 Mar 23;10:1027481. doi: 10.3389/fnut.2023.1027481 (PMC10076586; doi:10.3389/fnut.2023.1027481)
Supplement: Supplementary file 4 [file Table_4.DOCX]

Supplementary Table S4 Characteristics of the instruments for fresh fruit intake and their associations with low back pain.

| **SNP** | **Chr** | **Position** | **EA** | **OA** | **Exposure effect** |  |  |  | **Outcome effect** |  |  |
| --- | --- | --- | --- | --- | --- | --- | --- | --- | --- | --- | --- |
|  |  |  |  |  | **β** | **SE** | ***P*** |  | **β** | **SE** | ***P*** |
| rs10064431 | 5 | 92950673 | C | T | -0.008 | 0.001 | 6.00E-10 |  | -0.002 | 0.014 | 0.915 |
| rs10192394 | 2 | 146298007 | T | C | -0.008 | 0.001 | 4.50E-10 |  | 0.002 | 0.015 | 0.877 |
| rs10249294 | 7 | 143723137 | A | G | 0.020 | 0.001 | 4.10E-54 |  | 0.020 | 0.014 | 0.164 |
| rs10271924 | 7 | 153495206 | T | C | -0.007 | 0.001 | 2.00E-08 |  | 0.020 | 0.014 | 0.141 |
| rs1051547 | 16 | 19279380 | C | T | -0.008 | 0.001 | 1.10E-09 |  | 0.005 | 0.014 | 0.737 |
| rs10828266 | 10 | 22098701 | G | A | 0.012 | 0.001 | 8.10E-20 |  | -0.055 | 0.015 | 2.711E-04 |
| rs10838724 | 11 | 47527052 | T | G | 0.009 | 0.001 | 2.10E-12 |  | -0.008 | 0.015 | 0.562 |
| rs10840126 | 11 | 8825774 | G | A | -0.008 | 0.001 | 1.90E-09 |  | 0.002 | 0.014 | 0.869 |
| rs11032362 | 11 | 33759092 | A | G | 0.012 | 0.002 | 5.30E-09 |  | -0.047 | 0.030 | 0.119 |
| rs11085749 | 19 | 10961273 | A | G | -0.008 | 0.001 | 7.10E-10 |  | 0.005 | 0.014 | 0.721 |
| rs11248509 | 10 | 125134393 | T | A | 0.007 | 0.001 | 7.40E-09 |  | 0.001 | 0.014 | 0.921 |
| rs11896330 | 2 | 60235568 | A | G | -0.008 | 0.001 | 3.40E-11 |  | 0.002 | 0.015 | 0.895 |
| rs12044599 | 1 | 204564714 | G | A | 0.009 | 0.002 | 3.70E-10 |  | -0.012 | 0.016 | 0.444 |
| rs12536253 | 7 | 127595077 | C | G | -0.008 | 0.001 | 8.30E-09 |  | 0.030 | 0.018 | 0.089 |
| rs12641371 | 4 | 59882235 | T | C | 0.008 | 0.001 | 1.40E-10 |  | -0.015 | 0.014 | 0.289 |
| rs12780952 | 10 | 107577033 | A | G | 0.007 | 0.001 | 3.40E-08 |  | -0.017 | 0.015 | 0.253 |
| rs12885598 | 14 | 32071665 | A | G | 0.008 | 0.001 | 1.70E-09 |  | 0.023 | 0.014 | 0.091 |
| rs13072255 | 3 | 21038260 | C | A | 0.009 | 0.001 | 2.10E-13 |  | -0.021 | 0.014 | 0.138 |
| rs1356292 | 3 | 185824903 | T | C | 0.009 | 0.002 | 3.50E-09 |  | 0.022 | 0.017 | 0.2 |
| rs1375566 | 3 | 85642479 | A | G | -0.008 | 0.001 | 6.10E-10 |  | 0.003 | 0.015 | 0.848 |
| rs139042899 | 17 | 58443095 | C | A | 0.036 | 0.006 | 3.20E-09 |  | -0.027 | 0.053 | 0.607 |
| rs149449 | 5 | 95902093 | A | G | 0.007 | 0.001 | 2.40E-09 |  | -0.007 | 0.014 | 0.633 |
| rs1620977 | 1 | 72729142 | G | A | -0.013 | 0.001 | 1.10E-21 |  | 0.008 | 0.015 | 0.592 |
| rs17049185 | 2 | 58072660 | T | G | 0.008 | 0.001 | 7.30E-09 |  | -0.034 | 0.016 | 0.026 |
| rs1866823 | 8 | 57436577 | A | G | 0.007 | 0.001 | 2.10E-09 |  | -0.007 | 0.014 | 0.597 |
| rs2093654 | 9 | 5780121 | G | A | 0.007 | 0.001 | 1.50E-08 |  | 0.022 | 0.014 | 0.102 |
| rs2143081 | 6 | 50782834 | A | G | 0.008 | 0.001 | 1.30E-11 |  | -0.011 | 0.014 | 0.422 |
| rs2790688 | 1 | 153992909 | T | C | 0.011 | 0.002 | 1.50E-11 |  | -0.006 | 0.017 | 0.750 |
| rs28479795 | 14 | 79943606 | T | C | 0.011 | 0.001 | 2.50E-14 |  | 0.007 | 0.016 | 0.637 |
| rs2867113 | 2 | 651365 | A | G | -0.014 | 0.002 | 1.50E-12 |  | 0.019 | 0.018 | 0.293 |
| rs329274 | 7 | 35078743 | G | A | 0.007 | 0.001 | 2.80E-08 |  | -0.010 | 0.014 | 0.445 |
| rs34162196 | 14 | 22038125 | T | C | -0.018 | 0.002 | 4.00E-19 |  | 0.037 | 0.024 | 0.119 |
| rs4302893 | 9 | 1734863 | A | G | 0.007 | 0.001 | 1.30E-08 |  | -0.006 | 0.014 | 0.647 |
| rs4953150 | 2 | 45157336 | T | C | -0.008 | 0.001 | 6.60E-11 |  | 3.00E-04 | 0.014 | 0.981 |
| rs559734 | 1 | 97304868 | C | G | 0.008 | 0.001 | 1.10E-08 |  | 0.031 | 0.014 | 0.025 |
| rs586346 | 6 | 31875712 | C | T | -0.007 | 0.001 | 4.50E-08 |  | -0.018 | 0.015 | 0.225 |
| rs60452247 | 11 | 63981507 | A | G | 0.008 | 0.001 | 3.40E-10 |  | -0.009 | 0.015 | 0.532 |
| rs6475724 | 9 | 23274223 | T | C | 0.008 | 0.001 | 1.90E-08 |  | -0.025 | 0.015 | 0.098 |
| rs72974263 | 2 | 225447371 | T | C | 0.007 | 0.001 | 1.80E-08 |  | 0.013 | 0.015 | 0.361 |
| rs73455661 | 18 | 57968685 | G | A | 0.010 | 0.001 | 4.10E-14 |  | 0.016 | 0.016 | 0.305 |
| rs739320 | 19 | 49261368 | C | T | -0.009 | 0.001 | 1.90E-12 |  | 0.036 | 0.015 | 0.014 |
| rs7554485 | 1 | 65945906 | C | T | -0.008 | 0.001 | 1.70E-10 |  | 0.002 | 0.014 | 0.870 |
| rs7818437 | 8 | 10209623 | C | T | -0.008 | 0.001 | 3.00E-08 |  | 0.002 | 0.019 | 0.916 |
| rs7869969 | 9 | 96217447 | G | A | 0.008 | 0.001 | 5.70E-09 |  | 9.00E-04 | 0.014 | 0.950 |
| rs7982441 | 13 | 55924013 | C | T | -0.008 | 0.001 | 9.80E-10 |  | 0.015 | 0.016 | 0.351 |
| rs8095324 | 18 | 24131659 | G | A | -0.007 | 0.001 | 2.70E-08 |  | 0.002 | 0.014 | 0.915 |
| rs817223 | 2 | 104094008 | C | T | -0.007 | 0.001 | 2.80E-09 |  | -0.007 | 0.014 | 0.597 |
| rs862227 | 16 | 73602926 | G | A | -0.010 | 0.001 | 1.10E-16 |  | -0.012 | 0.014 | 0.372 |
| rs9517948 | 13 | 100650708 | T | C | 0.007 | 0.001 | 1.70E-08 |  | -0.013 | 0.014 | 0.366 |
| rs9919429 | 10 | 65313819 | G | A | -0.007 | 0.001 | 3.80E-08 |  | -0.012 | 0.014 | 0.384 |
| rs994270 | 6 | 51187787 | G | C | 0.013 | 0.001 | 4.20E-20 |  | -0.013 | 0.018 | 0.452 |

EA, effect allele; OA, other allele; SNP, single nucleotide polymorphism; SE, standard error.
