# Supplementary material for: Dried fruit intake causally protects against low back pain: A Mendelian randomization study
Source: Front Nutr. 2023 Mar 23;10:1027481. doi: 10.3389/fnut.2023.1027481 (PMC10076586; doi:10.3389/fnut.2023.1027481)
Supplement: Supplementary file 5 [file Table_5.DOCX]

Supplementary Table S5 Characteristics of the instruments for BMI and their associations with low back pain.

| **SNP** | **Chr** | **Position** | **EA** | **OA** | **Exposure effect** |  |  |  | **Outcome effect** |  |  |
| --- | --- | --- | --- | --- | --- | --- | --- | --- | --- | --- | --- |
|  |  |  |  |  | **β** | **SE** | ***P*** |  | **β** | **SE** | ***P*** |
| rs10009336 | 4 | 44480783 | T | C | -0.014 | 0.002 | 2.20E-10 |  | -0.017 | 0.018 | 0.366 |
| rs10132280 | 14 | 25928179 | A | C | -0.022 | 0.002 | 5.60E-35 |  | 0.002 | 0.014 | 0.907 |
| rs10169594 | 2 | 41637688 | C | T | 0.012 | 0.002 | 2.00E-11 |  | -0.005 | 0.014 | 0.718 |
| rs10182181 | 2 | 25150296 | G | A | 0.033 | 0.002 | 6.70E-90 |  | 0.019 | 0.014 | 0.167 |
| rs10192119 | 2 | 164581241 | G | T | 0.017 | 0.002 | 3.00E-14 |  | 0.008 | 0.017 | 0.632 |
| rs10197031 | 2 | 105454590 | C | T | 0.017 | 0.002 | 1.90E-18 |  | 0.005 | 0.014 | 0.711 |
| rs10247983 | 7 | 114590228 | A | G | 0.020 | 0.003 | 1.70E-09 |  | -0.022 | 0.024 | 0.373 |
| rs10248136 | 7 | 39077397 | T | C | -0.010 | 0.002 | 2.00E-08 |  | -0.009 | 0.014 | 0.506 |
| rs10269783 | 7 | 49616203 | A | G | 0.013 | 0.002 | 1.40E-15 |  | -0.019 | 0.014 | 0.156 |
| rs10408324 | 19 | 51774806 | T | C | -0.012 | 0.002 | 9.50E-11 |  | -0.021 | 0.015 | 0.161 |
| rs10478110 | 5 | 112445734 | C | A | 0.01 | 0.002 | 9.60E-09 |  | -0.008 | 0.014 | 0.548 |
| rs1048932 | 11 | 115044850 | A | C | -0.016 | 0.002 | 3.80E-22 |  | -0.017 | 0.014 | 0.217 |
| rs10492229 | 12 | 110602173 | T | C | 0.014 | 0.002 | 7.70E-14 |  | 0.018 | 0.015 | 0.244 |
| rs10510419 | 3 | 12426936 | T | G | -0.018 | 0.002 | 2.20E-14 |  | 0.029 | 0.019 | 0.126 |
| rs10518694 | 15 | 53072673 | A | C | 0.015 | 0.003 | 3.30E-09 |  | 0.023 | 0.022 | 0.290 |
| rs1064213 | 2 | 198950240 | A | G | 0.012 | 0.002 | 2.40E-12 |  | 0.028 | 0.014 | 0.045 |
| rs10733051 | 1 | 167280354 | G | A | -0.010 | 0.002 | 2.90E-09 |  | 0.017 | 0.014 | 0.222 |
| rs10742752 | 11 | 45438374 | C | T | 0.012 | 0.002 | 1.10E-13 |  | -0.002 | 0.014 | 0.862 |
| rs10747488 | 1 | 98299475 | A | C | -0.012 | 0.002 | 1.20E-09 |  | -0.023 | 0.015 | 0.113 |
| rs10750215 | 11 | 122505344 | T | G | 0.011 | 0.002 | 1.30E-10 |  | -0.016 | 0.014 | 0.256 |
| rs1075901 | 17 | 15943910 | C | T | 0.012 | 0.002 | 1.20E-13 |  | 0.010 | 0.014 | 0.466 |
| rs10768994 | 11 | 43936945 | C | T | -0.011 | 0.002 | 6.40E-12 |  | 0.017 | 0.014 | 0.222 |
| rs10795422 | 10 | 16759312 | G | A | 0.014 | 0.002 | 9.30E-14 |  | -0.009 | 0.015 | 0.532 |
| rs10811871 | 9 | 23200766 | G | A | -0.011 | 0.002 | 1.60E-09 |  | 0.015 | 0.014 | 0.294 |
| rs10858334 | 9 | 137989785 | G | C | 0.014 | 0.003 | 2.70E-08 |  | 0.025 | 0.026 | 0.328 |
| rs10867256 | 9 | 81367391 | T | C | -0.012 | 0.002 | 8.70E-12 |  | -0.029 | 0.014 | 0.039 |
| rs10878946 | 12 | 69642315 | T | C | -0.014 | 0.002 | 3.60E-13 |  | -0.020 | 0.014 | 0.148 |
| rs10914462 | 1 | 32125943 | G | A | -0.011 | 0.002 | 1.50E-10 |  | -0.006 | 0.014 | 0.665 |
| rs10915840 | 1 | 225668524 | A | G | -0.012 | 0.002 | 1.30E-09 |  | -0.015 | 0.016 | 0.342 |
| rs10920678 | 1 | 190239907 | G | A | -0.016 | 0.002 | 1.50E-21 |  | 0.002 | 0.014 | 0.867 |
| rs10938397 | 4 | 45182527 | G | A | 0.032 | 0.002 | 3.40E-86 |  | 0.025 | 0.014 | 0.064 |
| rs10942267 | 5 | 80841914 | G | A | -0.016 | 0.002 | 3.90E-17 |  | -0.007 | 0.016 | 0.651 |
| rs10953740 | 7 | 113460282 | G | A | -0.015 | 0.002 | 1.00E-18 |  | -0.019 | 0.014 | 0.170 |
| rs10962550 | 9 | 16720329 | C | G | 0.018 | 0.002 | 6.20E-16 |  | -0.012 | 0.018 | 0.487 |
| rs10968114 | 9 | 27800007 | C | A | -0.011 | 0.002 | 6.10E-11 |  | 0.024 | 0.014 | 0.073 |
| rs10971709 | 9 | 33804813 | T | C | 0.013 | 0.002 | 6.20E-10 |  | 0.004 | 0.018 | 0.839 |
| rs10984756 | 9 | 122651784 | G | C | 0.017 | 0.003 | 1.10E-09 |  | 0.022 | 0.025 | 0.377 |
| rs11030618 | 11 | 29243293 | T | C | 0.011 | 0.002 | 2.40E-10 |  | 0.004 | 0.014 | 0.745 |
| rs11066188 | 12 | 112610714 | A | G | -0.012 | 0.002 | 8.10E-13 |  | -0.014 | 0.014 | 0.316 |
| rs11084553 | 19 | 31019780 | G | A | -0.021 | 0.002 | 1.80E-18 |  | 0.048 | 0.020 | 0.016 |
| rs11105839 | 12 | 91237920 | A | T | -0.011 | 0.002 | 1.10E-10 |  | -0.023 | 0.014 | 0.106 |
| rs11115176 | 12 | 82465797 | C | T | -0.012 | 0.002 | 2.00E-10 |  | -0.035 | 0.016 | 0.032 |
| rs11118308 | 1 | 219633869 | G | A | -0.010 | 0.002 | 4.80E-10 |  | 0.029 | 0.014 | 0.033 |
| rs1112613 | 13 | 53651850 | A | G | -0.013 | 0.002 | 3.40E-09 |  | -0.036 | 0.017 | 0.036 |
| rs11150911 | 18 | 73498528 | C | A | -0.013 | 0.002 | 4.70E-13 |  | 0.013 | 0.015 | 0.367 |
| rs11165643 | 1 | 96924097 | T | C | 0.021 | 0.002 | 1.40E-35 |  | 0.009 | 0.014 | 0.536 |
| rs11170468 | 12 | 39430048 | C | A | -0.012 | 0.002 | 1.90E-10 |  | -0.010 | 0.017 | 0.556 |
| rs11173522 | 12 | 60953472 | A | C | 0.013 | 0.002 | 1.10E-09 |  | 0.002 | 0.017 | 0.890 |
| rs11185111 | 1 | 107962328 | A | G | -0.013 | 0.002 | 7.70E-12 |  | 0.008 | 0.014 | 0.563 |
| rs11251352 | 10 | 2585792 | G | A | 0.011 | 0.002 | 7.00E-10 |  | -0.005 | 0.014 | 0.692 |
| rs11496125 | 7 | 103417557 | T | C | 0.017 | 0.002 | 3.00E-22 |  | 0.025 | 0.014 | 0.066 |
| rs11505821 | 7 | 76818677 | T | A | 0.031 | 0.004 | 2.70E-19 |  | -0.027 | 0.028 | 0.346 |
| rs11538 | 22 | 18220831 | G | A | 0.014 | 0.002 | 3.30E-09 |  | -0.030 | 0.017 | 0.083 |
| rs1158805 | 18 | 40736590 | A | C | -0.014 | 0.002 | 1.20E-14 |  | 0.003 | 0.014 | 0.843 |
| rs11609659 | 12 | 108296260 | C | T | -0.015 | 0.002 | 2.20E-14 |  | -0.014 | 0.016 | 0.364 |
| rs11611246 | 12 | 939480 | T | G | 0.024 | 0.002 | 5.00E-32 |  | -0.002 | 0.017 | 0.923 |
| rs11615578 | 12 | 121714935 | T | C | 0.013 | 0.002 | 8.10E-11 |  | -0.005 | 0.016 | 0.755 |
| rs11656076 | 17 | 31464270 | A | G | -0.014 | 0.002 | 5.60E-12 |  | -0.009 | 0.015 | 0.542 |
| rs11672660 | 19 | 46180184 | T | C | -0.034 | 0.002 | 1.70E-60 |  | -0.007 | 0.016 | 0.664 |
| rs11713193 | 3 | 49924424 | A | G | 0.024 | 0.002 | 2.40E-44 |  | 0.037 | 0.014 | 0.008 |
| rs11736228 | 4 | 147376805 | T | A | -0.014 | 0.002 | 4.10E-12 |  | 0.014 | 0.016 | 0.398 |
| rs11738695 | 5 | 108699161 | A | C | 0.010 | 0.002 | 2.00E-08 |  | -0.003 | 0.014 | 0.841 |
| rs11739877 | 5 | 105876806 | T | C | 0.012 | 0.002 | 6.60E-11 |  | -0.015 | 0.014 | 0.266 |
| rs11781699 | 8 | 118863061 | C | T | 0.013 | 0.002 | 3.10E-10 |  | -0.007 | 0.015 | 0.647 |
| rs1187352 | 9 | 87293457 | C | T | 0.012 | 0.002 | 6.00E-11 |  | -0.002 | 0.015 | 0.876 |
| rs11880870 | 19 | 18830704 | G | A | -0.019 | 0.002 | 1.00E-28 |  | -0.006 | 0.014 | 0.666 |
| rs11889536 | 2 | 220163543 | G | A | -0.019 | 0.002 | 6.40E-15 |  | -0.082 | 0.031 | 0.008 |
| rs11908637 | 20 | 47428485 | A | G | -0.012 | 0.002 | 4.90E-09 |  | -0.003 | 0.015 | 0.844 |
| rs11945861 | 4 | 65700865 | A | G | -0.015 | 0.002 | 5.00E-13 |  | 0.010 | 0.016 | 0.531 |
| rs11951673 | 5 | 95861012 | T | C | -0.012 | 0.002 | 1.10E-13 |  | 0.031 | 0.015 | 0.037 |
| rs12041258 | 1 | 195047936 | C | T | -0.015 | 0.002 | 9.50E-13 |  | -0.014 | 0.016 | 0.379 |
| rs12044597 | 1 | 1708801 | G | A | 0.014 | 0.002 | 1.70E-18 |  | -0.016 | 0.014 | 0.239 |
| rs12049202 | 1 | 77967523 | T | C | 0.024 | 0.002 | 1.00E-28 |  | 0.011 | 0.015 | 0.451 |
| rs12098284 | 10 | 76047464 | T | C | 0.018 | 0.003 | 1.80E-11 |  | 0.005 | 0.026 | 0.851 |
| rs12150665 | 17 | 34914787 | C | T | -0.016 | 0.002 | 1.60E-22 |  | -0.019 | 0.014 | 0.176 |
| rs1218822 | 13 | 28011963 | A | G | 0.017 | 0.002 | 1.90E-22 |  | 0.022 | 0.014 | 0.118 |
| rs12299814 | 12 | 90216146 | A | C | -0.016 | 0.002 | 5.20E-15 |  | 0.001 | 0.014 | 0.925 |
| rs12328930 | 2 | 175079125 | C | T | 0.010 | 0.002 | 1.80E-08 |  | 0.005 | 0.014 | 0.746 |
| rs12334877 | 8 | 67194171 | A | G | -0.014 | 0.002 | 7.70E-11 |  | -0.023 | 0.016 | 0.156 |
| rs12364470 | 11 | 134601012 | G | T | 0.018 | 0.002 | 1.10E-15 |  | 0.014 | 0.016 | 0.400 |
| rs12369179 | 12 | 122963550 | T | C | -0.036 | 0.003 | 2.50E-31 |  | -0.021 | 0.024 | 0.400 |
| rs12416812 | 11 | 888632 | A | G | 0.011 | 0.002 | 6.10E-12 |  | -0.002 | 0.014 | 0.870 |
| rs1241986 | 18 | 6873954 | A | G | -0.014 | 0.002 | 1.10E-08 |  | -0.014 | 0.018 | 0.435 |
| rs12422552 | 12 | 14413931 | C | G | -0.013 | 0.002 | 1.60E-11 |  | -0.019 | 0.017 | 0.252 |
| rs12429545 | 13 | 54102206 | A | G | 0.032 | 0.003 | 9.60E-38 |  | 0.042 | 0.020 | 0.034 |
| rs12448257 | 16 | 3599655 | A | G | 0.018 | 0.002 | 8.10E-20 |  | -0.034 | 0.018 | 0.068 |
| rs12564992 | 1 | 174478100 | G | A | 0.020 | 0.003 | 5.30E-14 |  | 0.038 | 0.025 | 0.127 |
| rs12593036 | 15 | 81058652 | G | A | -0.015 | 0.002 | 3.80E-16 |  | 0.008 | 0.015 | 0.620 |
| rs12602912 | 17 | 65870073 | T | C | 0.018 | 0.002 | 9.90E-18 |  | 0.037 | 0.016 | 0.020 |
| rs1260326 | 2 | 27730940 | C | T | 0.011 | 0.002 | 3.90E-10 |  | -0.013 | 0.014 | 0.350 |
| rs12629015 | 3 | 119618053 | G | A | -0.014 | 0.002 | 2.10E-09 |  | 0.010 | 0.015 | 0.527 |
| rs1266874 | 6 | 51779638 | G | A | 0.014 | 0.002 | 9.80E-15 |  | -0.018 | 0.014 | 0.176 |
| rs12675063 | 8 | 132879047 | T | A | 0.016 | 0.003 | 1.30E-09 |  | -0.036 | 0.027 | 0.183 |
| rs1268065 | 6 | 126042783 | A | G | -0.010 | 0.002 | 1.00E-09 |  | 0.006 | 0.014 | 0.648 |
| rs12680842 | 8 | 95582606 | G | A | -0.013 | 0.002 | 4.40E-14 |  | -0.015 | 0.014 | 0.269 |
| rs12718572 | 7 | 50573325 | T | C | -0.012 | 0.002 | 3.00E-11 |  | -0.018 | 0.015 | 0.229 |
| rs12762034 | 10 | 33969931 | C | T | 0.024 | 0.003 | 7.30E-14 |  | -0.012 | 0.023 | 0.590 |
| rs12779328 | 10 | 12943973 | T | C | 0.011 | 0.002 | 4.50E-08 |  | 0.018 | 0.016 | 0.265 |
| rs1285997 | 14 | 91513029 | G | C | 0.014 | 0.002 | 1.20E-13 |  | 0.023 | 0.015 | 0.137 |
| rs12888545 | 14 | 88308044 | G | A | 0.014 | 0.002 | 9.10E-12 |  | -0.008 | 0.016 | 0.616 |
| rs12888955 | 14 | 103256877 | A | G | -0.018 | 0.002 | 1.40E-22 |  | -0.039 | 0.014 | 0.005 |
| rs12905439 | 15 | 99521883 | G | C | -0.012 | 0.002 | 1.40E-10 |  | -0.015 | 0.014 | 0.289 |
| rs12914489 | 15 | 74187937 | A | G | 0.017 | 0.003 | 3.80E-10 |  | 0.024 | 0.020 | 0.216 |
| rs12922346 | 16 | 82438337 | C | G | 0.014 | 0.002 | 1.00E-11 |  | 0.002 | 0.017 | 0.924 |
| rs12933482 | 16 | 72189604 | G | A | 0.019 | 0.003 | 4.90E-11 |  | 0.003 | 0.029 | 0.904 |
| rs12936083 | 17 | 4801887 | G | A | 0.014 | 0.002 | 4.10E-13 |  | 0.024 | 0.014 | 0.075 |
| rs12939549 | 17 | 78611724 | G | A | -0.018 | 0.002 | 2.70E-28 |  | 0.018 | 0.014 | 0.213 |
| rs1296328 | 4 | 137083193 | C | A | -0.018 | 0.002 | 4.90E-24 |  | 0.014 | 0.014 | 0.287 |
| rs12981256 | 19 | 1865901 | A | G | 0.014 | 0.002 | 1.10E-15 |  | -0.008 | 0.014 | 0.584 |
| rs13021737 | 2 | 632348 | G | A | 0.057 | 0.002 | 7.50E-157 |  | -0.019 | 0.018 | 0.294 |
| rs13069244 | 3 | 180441172 | A | G | 0.019 | 0.003 | 3.00E-09 |  | 0.022 | 0.032 | 0.487 |
| rs13107325 | 4 | 103188709 | T | C | 0.047 | 0.003 | 1.10E-47 |  | 0.106 | 0.058 | 0.069 |
| rs13110266 | 4 | 162129844 | A | G | -0.012 | 0.002 | 1.90E-12 |  | -0.005 | 0.014 | 0.741 |
| rs13147390 | 4 | 80712000 | C | T | 0.010 | 0.002 | 1.00E-08 |  | -0.011 | 0.015 | 0.457 |
| rs13174863 | 5 | 139080745 | G | A | 0.019 | 0.002 | 2.90E-16 |  | 0.005 | 0.019 | 0.786 |
| rs13184896 | 5 | 122734005 | T | G | -0.013 | 0.002 | 3.30E-16 |  | -0.013 | 0.014 | 0.334 |
| rs13191362 | 6 | 163033350 | G | A | -0.024 | 0.003 | 5.90E-21 |  | -0.052 | 0.028 | 0.068 |
| rs1320903 | 3 | 131758077 | A | G | 0.022 | 0.002 | 9.20E-32 |  | 0.009 | 0.015 | 0.543 |
| rs1321432 | 20 | 6614691 | C | A | 0.020 | 0.002 | 3.50E-29 |  | -0.014 | 0.014 | 0.322 |
| rs13240600 | 7 | 99064466 | G | A | -0.020 | 0.002 | 3.50E-17 |  | 0.016 | 0.016 | 0.326 |
| rs13250058 | 8 | 112270826 | T | G | 0.011 | 0.002 | 2.90E-10 |  | -0.014 | 0.015 | 0.330 |
| rs13263601 | 8 | 14095900 | C | A | 0.015 | 0.002 | 2.20E-17 |  | 0.005 | 0.015 | 0.764 |
| rs1327259 | 6 | 51177811 | G | A | -0.016 | 0.002 | 1.70E-18 |  | 0.004 | 0.014 | 0.764 |
| rs13287131 | 9 | 92119579 | C | T | 0.012 | 0.002 | 6.80E-10 |  | -0.001 | 0.015 | 0.936 |
| rs13329567 | 15 | 68104367 | T | C | -0.029 | 0.002 | 1.00E-50 |  | -0.025 | 0.018 | 0.179 |
| rs1365466 | 18 | 36182440 | T | C | -0.014 | 0.002 | 3.30E-13 |  | 0.010 | 0.017 | 0.542 |
| rs1371108 | 2 | 81816251 | A | C | 0.012 | 0.002 | 9.00E-11 |  | -0.012 | 0.015 | 0.413 |
| rs1409818 | 20 | 21381121 | T | C | 0.020 | 0.003 | 2.50E-12 |  | -0.009 | 0.027 | 0.732 |
| rs1412235 | 9 | 28410996 | C | G | 0.025 | 0.002 | 6.00E-45 |  | 0.006 | 0.014 | 0.659 |
| rs1421334 | 8 | 30865733 | C | A | -0.013 | 0.002 | 1.00E-12 |  | 0.009 | 0.014 | 0.529 |
| rs1430387 | 18 | 58227112 | C | T | -0.011 | 0.002 | 5.80E-11 |  | 0.016 | 0.014 | 0.240 |
| rs1431659 | 8 | 73439070 | G | A | -0.020 | 0.002 | 6.00E-24 |  | 0.003 | 0.016 | 0.839 |
| rs1436344 | 3 | 104606144 | C | G | 0.014 | 0.002 | 4.10E-16 |  | 0.019 | 0.014 | 0.186 |
| rs1445652 | 2 | 155668460 | A | G | 0.012 | 0.002 | 4.30E-08 |  | -0.017 | 0.020 | 0.394 |
| rs1452075 | 3 | 62481063 | T | C | 0.014 | 0.002 | 1.30E-14 |  | -0.052 | 0.015 | 0.001 |
| rs1465900 | 11 | 76473138 | C | A | -0.013 | 0.002 | 4.80E-10 |  | -0.046 | 0.017 | 0.006 |
| rs1472169 | 9 | 37209396 | T | C | -0.014 | 0.002 | 2.80E-15 |  | -0.023 | 0.015 | 0.124 |
| rs1477199 | 16 | 53712135 | G | A | 0.023 | 0.002 | 9.40E-22 |  | 0.009 | 0.021 | 0.678 |
| rs1492767 | 4 | 55221467 | T | C | 0.009 | 0.002 | 1.00E-08 |  | 0.012 | 0.014 | 0.398 |
| rs1503526 | 5 | 63020706 | C | T | 0.014 | 0.002 | 5.50E-17 |  | 0.006 | 0.014 | 0.656 |
| rs1522569 | 4 | 171632637 | G | T | -0.016 | 0.002 | 2.90E-13 |  | -0.013 | 0.025 | 0.599 |
| rs1528435 | 2 | 181550962 | T | C | 0.016 | 0.002 | 9.10E-23 |  | 0.021 | 0.014 | 0.131 |
| rs1535660 | 9 | 10371073 | C | T | -0.015 | 0.003 | 5.20E-09 |  | -0.027 | 0.022 | 0.215 |
| rs1538247 | 6 | 153395344 | C | T | 0.011 | 0.002 | 1.00E-08 |  | 0.026 | 0.015 | 0.071 |
| rs156201 | 6 | 104847441 | C | G | 0.012 | 0.002 | 5.80E-10 |  | -0.006 | 0.014 | 0.648 |
| rs1656377 | 3 | 158285280 | C | T | 0.010 | 0.002 | 1.60E-08 |  | -0.011 | 0.014 | 0.407 |
| rs1681740 | 10 | 118564313 | C | A | -0.012 | 0.002 | 1.10E-10 |  | 0.019 | 0.014 | 0.181 |
| rs16849710 | 1 | 202106797 | G | A | -0.012 | 0.002 | 6.00E-11 |  | -0.017 | 0.014 | 0.217 |
| rs16851483 | 3 | 141275436 | T | G | 0.037 | 0.004 | 3.20E-26 |  | 0.006 | 0.031 | 0.857 |
| rs16871902 | 5 | 3488462 | A | G | 0.013 | 0.002 | 4.60E-13 |  | 0.017 | 0.014 | 0.214 |
| rs16903285 | 5 | 87978252 | C | T | 0.033 | 0.003 | 7.60E-38 |  | 0.008 | 0.022 | 0.729 |
| rs17001561 | 4 | 77096118 | A | G | 0.015 | 0.002 | 3.80E-11 |  | -0.022 | 0.021 | 0.289 |
| rs17014375 | 1 | 209543560 | G | T | 0.017 | 0.003 | 1.10E-11 |  | 0.012 | 0.022 | 0.585 |
| rs17033117 | 3 | 35443653 | T | C | 0.014 | 0.002 | 8.90E-10 |  | 0.030 | 0.018 | 0.101 |
| rs17056301 | 5 | 158271680 | C | T | 0.012 | 0.002 | 2.40E-09 |  | 0.022 | 0.016 | 0.179 |
| rs17113297 | 10 | 102395982 | T | C | 0.017 | 0.002 | 2.10E-15 |  | -0.026 | 0.016 | 0.109 |
| rs17119937 | 8 | 14502274 | C | T | 0.021 | 0.004 | 5.60E-09 |  | -0.016 | 0.030 | 0.593 |
| rs17203016 | 2 | 208255518 | G | A | 0.015 | 0.002 | 2.10E-13 |  | 0.055 | 0.017 | 0.001 |
| rs17207196 | 7 | 75101065 | T | C | -0.022 | 0.002 | 2.10E-35 |  | 0.000 | 0.014 | 0.992 |
| rs17238110 | 15 | 62150364 | G | A | -0.035 | 0.005 | 2.00E-12 |  | -0.022 | 0.022 | 0.325 |
| rs17311369 | 15 | 47709199 | T | C | -0.010 | 0.002 | 3.10E-08 |  | 0.028 | 0.015 | 0.058 |
| rs17399237 | 2 | 35471626 | C | T | -0.013 | 0.002 | 6.70E-14 |  | -0.022 | 0.014 | 0.117 |
| rs17405819 | 8 | 76806584 | C | T | -0.022 | 0.002 | 4.30E-33 |  | -0.004 | 0.015 | 0.792 |
| rs17424296 | 5 | 60838903 | A | G | -0.011 | 0.002 | 2.40E-09 |  | 0.017 | 0.014 | 0.230 |
| rs17425707 | 1 | 57874879 | C | T | 0.017 | 0.003 | 4.40E-09 |  | 0.029 | 0.027 | 0.278 |
| rs17446257 | 13 | 40749213 | A | G | 0.015 | 0.003 | 2.90E-09 |  | -0.011 | 0.019 | 0.566 |
| rs17499593 | 2 | 172649755 | G | C | 0.013 | 0.002 | 1.10E-08 |  | 0.050 | 0.020 | 0.014 |
| rs17513613 | 19 | 30286822 | C | T | 0.019 | 0.002 | 3.60E-26 |  | 0.009 | 0.014 | 0.510 |
| rs175165 | 22 | 20116015 | G | T | -0.010 | 0.002 | 5.20E-09 |  | -0.023 | 0.014 | 0.091 |
| rs17535749 | 3 | 10027724 | A | G | 0.015 | 0.003 | 2.50E-08 |  | 0.029 | 0.024 | 0.228 |
| rs17551974 | 2 | 142293146 | A | C | -0.014 | 0.002 | 1.90E-10 |  | -0.016 | 0.017 | 0.326 |
| rs17636031 | 10 | 126594078 | C | T | 0.016 | 0.002 | 1.20E-17 |  | -0.020 | 0.019 | 0.292 |
| rs17663412 | 5 | 167595121 | A | C | 0.016 | 0.003 | 6.10E-09 |  | 0.097 | 0.025 | 0.000 |
| rs17710386 | 18 | 63461201 | C | T | 0.013 | 0.002 | 1.00E-12 |  | -0.002 | 0.014 | 0.870 |
| rs17724992 | 19 | 18454825 | G | A | -0.018 | 0.002 | 1.00E-22 |  | -0.015 | 0.016 | 0.342 |
| rs17789218 | 6 | 100600097 | C | T | 0.013 | 0.002 | 7.40E-12 |  | -0.011 | 0.017 | 0.503 |
| rs17806379 | 20 | 51107290 | T | C | -0.026 | 0.002 | 1.50E-30 |  | -0.012 | 0.019 | 0.514 |
| rs1784460 | 11 | 118938371 | A | T | 0.013 | 0.002 | 9.00E-14 |  | -0.019 | 0.015 | 0.196 |
| rs1804528 | 4 | 146056320 | A | G | 0.011 | 0.002 | 3.00E-08 |  | -0.001 | 0.014 | 0.956 |
| rs1830074 | 7 | 6718674 | C | T | 0.012 | 0.002 | 1.40E-09 |  | 0.003 | 0.015 | 0.852 |
| rs1836303 | 15 | 46539116 | G | A | 0.012 | 0.002 | 5.30E-11 |  | 0.016 | 0.015 | 0.267 |
| rs1863652 | 4 | 95991417 | A | G | -0.012 | 0.002 | 1.40E-10 |  | 0.027 | 0.015 | 0.070 |
| rs1884389 | 20 | 1410582 | T | C | -0.010 | 0.002 | 4.00E-09 |  | -0.002 | 0.014 | 0.897 |
| rs1885728 | 6 | 5977833 | A | G | 0.011 | 0.002 | 1.00E-08 |  | -0.007 | 0.015 | 0.643 |
| rs1891216 | 1 | 7728391 | G | T | 0.011 | 0.002 | 2.40E-09 |  | -0.009 | 0.014 | 0.523 |
| rs1896767 | 16 | 62838304 | A | G | -0.011 | 0.002 | 2.40E-10 |  | 0.018 | 0.014 | 0.176 |
| rs1927790 | 13 | 96922191 | C | T | 0.015 | 0.002 | 1.80E-19 |  | 0.016 | 0.014 | 0.242 |
| rs1928295 | 9 | 120378483 | C | T | -0.014 | 0.002 | 5.40E-18 |  | -0.007 | 0.014 | 0.611 |
| rs1937683 | 10 | 53679060 | T | C | 0.011 | 0.002 | 3.20E-09 |  | -0.005 | 0.014 | 0.731 |
| rs1948080 | 9 | 11852043 | G | T | -0.014 | 0.002 | 1.10E-14 |  | -0.028 | 0.015 | 0.061 |
| rs1982441 | 8 | 28021769 | T | G | 0.018 | 0.003 | 7.00E-12 |  | 0.023 | 0.017 | 0.192 |
| rs1982725 | 19 | 30618771 | T | C | 0.010 | 0.002 | 3.30E-08 |  | 0.010 | 0.014 | 0.470 |
| rs1993709 | 1 | 72838529 | G | A | 0.033 | 0.002 | 1.90E-57 |  | 0.014 | 0.020 | 0.466 |
| rs2007231 | 1 | 115266306 | T | C | -0.010 | 0.002 | 5.20E-09 |  | 0.011 | 0.015 | 0.445 |
| rs200810 | 6 | 97922184 | C | T | -0.014 | 0.002 | 5.50E-16 |  | -0.012 | 0.014 | 0.407 |
| rs2009416 | 5 | 92415111 | T | C | -0.012 | 0.002 | 1.10E-11 |  | -0.017 | 0.014 | 0.212 |
| rs2051559 | 4 | 3298800 | C | T | 0.018 | 0.003 | 5.00E-12 |  | 0.045 | 0.022 | 0.040 |
| rs2065418 | 11 | 30422068 | G | T | -0.017 | 0.002 | 3.60E-20 |  | -0.002 | 0.015 | 0.896 |
| rs208015 | 17 | 46252346 | C | T | -0.036 | 0.003 | 1.40E-25 |  | 0.005 | 0.021 | 0.817 |
| rs2124499 | 3 | 123093541 | C | G | -0.012 | 0.002 | 3.40E-13 |  | -0.024 | 0.014 | 0.093 |
| rs2143253 | 20 | 41987392 | A | G | -0.019 | 0.003 | 1.10E-12 |  | -0.001 | 0.023 | 0.963 |
| rs215634 | 7 | 32369148 | G | A | -0.015 | 0.002 | 2.60E-17 |  | -0.011 | 0.015 | 0.454 |
| rs2162524 | 2 | 230817437 | C | T | 0.016 | 0.002 | 4.10E-17 |  | 0.010 | 0.015 | 0.510 |
| rs217671 | 14 | 62360464 | G | A | 0.014 | 0.002 | 1.30E-13 |  | 0.016 | 0.017 | 0.331 |
| rs2228213 | 6 | 12124855 | A | G | -0.014 | 0.002 | 4.60E-16 |  | 0.024 | 0.014 | 0.085 |
| rs2235564 | 1 | 6713114 | T | C | 0.013 | 0.002 | 3.70E-13 |  | -0.023 | 0.014 | 0.101 |
| rs2246012 | 6 | 131898208 | C | T | 0.016 | 0.002 | 3.10E-13 |  | 0.009 | 0.016 | 0.597 |
| rs2283093 | 7 | 126721231 | T | C | 0.013 | 0.002 | 3.10E-09 |  | 0.018 | 0.017 | 0.311 |
| rs2285178 | 22 | 38205989 | C | T | 0.011 | 0.002 | 9.40E-09 |  | 0.014 | 0.014 | 0.337 |
| rs2306537 | 12 | 133423695 | G | A | 0.013 | 0.002 | 8.70E-13 |  | -0.030 | 0.015 | 0.054 |
| rs2307111 | 5 | 75003678 | C | T | -0.027 | 0.002 | 1.60E-58 |  | -0.009 | 0.014 | 0.536 |
| rs2317299 | 2 | 236903093 | C | T | -0.011 | 0.002 | 1.30E-09 |  | -0.025 | 0.014 | 0.067 |
| rs2325036 | 3 | 85819412 | C | A | -0.018 | 0.002 | 3.60E-27 |  | -0.006 | 0.014 | 0.644 |
| rs2357760 | 6 | 120213880 | A | G | 0.015 | 0.002 | 6.80E-17 |  | 0.012 | 0.014 | 0.398 |
| rs2361988 | 16 | 398151 | C | T | -0.016 | 0.002 | 5.20E-15 |  | -0.001 | 0.017 | 0.941 |
| rs2365389 | 3 | 61236462 | T | C | -0.017 | 0.002 | 1.30E-25 |  | 0.009 | 0.014 | 0.513 |
| rs2367112 | 5 | 64168193 | G | T | -0.012 | 0.002 | 2.30E-13 |  | -0.007 | 0.014 | 0.600 |
| rs2423668 | 20 | 12430673 | C | T | -0.011 | 0.002 | 2.80E-08 |  | 0.015 | 0.014 | 0.265 |
| rs2425840 | 20 | 44904838 | C | A | 0.012 | 0.002 | 1.60E-11 |  | -0.005 | 0.014 | 0.717 |
| rs2429150 | 12 | 2152655 | C | A | 0.011 | 0.002 | 2.70E-10 |  | 0.020 | 0.014 | 0.143 |
| rs2479958 | 13 | 111984244 | G | A | -0.015 | 0.002 | 1.50E-17 |  | -0.008 | 0.014 | 0.566 |
| rs2481665 | 1 | 62594677 | C | T | -0.016 | 0.002 | 7.20E-23 |  | -0.002 | 0.014 | 0.869 |
| rs2543132 | 8 | 15536311 | C | G | 0.015 | 0.002 | 5.00E-11 |  | 0.014 | 0.019 | 0.457 |
| rs2600226 | 3 | 12928762 | T | C | -0.012 | 0.002 | 3.70E-10 |  | 0.005 | 0.014 | 0.738 |
| rs2605603 | 11 | 93221105 | A | G | -0.010 | 0.002 | 2.50E-10 |  | -0.048 | 0.014 | 0.000 |
| rs2608703 | 12 | 41846769 | A | C | 0.014 | 0.002 | 1.90E-16 |  | -0.001 | 0.014 | 0.919 |
| rs262130 | 6 | 142853486 | T | C | 0.013 | 0.002 | 1.80E-08 |  | -0.019 | 0.017 | 0.262 |
| rs2693826 | 2 | 6160943 | A | G | -0.014 | 0.002 | 2.00E-15 |  | -0.019 | 0.014 | 0.156 |
| rs2694047 | 8 | 116750548 | G | A | 0.019 | 0.002 | 3.90E-21 |  | -0.011 | 0.015 | 0.470 |
| rs273504 | 19 | 18215247 | G | A | 0.015 | 0.002 | 4.40E-18 |  | 0.014 | 0.014 | 0.304 |
| rs2744974 | 6 | 34579431 | T | C | 0.025 | 0.002 | 1.40E-45 |  | 0.069 | 0.014 | 0.000 |
| rs2791653 | 1 | 11129848 | G | A | -0.014 | 0.002 | 1.30E-13 |  | 0.004 | 0.016 | 0.790 |
| rs2820311 | 1 | 201841476 | G | A | 0.024 | 0.002 | 4.10E-38 |  | 0.007 | 0.015 | 0.658 |
| rs2832283 | 21 | 30690558 | A | G | 0.012 | 0.002 | 5.80E-09 |  | -0.010 | 0.015 | 0.529 |
| rs2836964 | 21 | 40631006 | C | T | -0.011 | 0.002 | 1.30E-09 |  | -0.009 | 0.016 | 0.579 |
| rs2861683 | 2 | 67836507 | C | A | -0.014 | 0.002 | 1.30E-16 |  | -0.014 | 0.014 | 0.298 |
| rs2868975 | 3 | 116935323 | A | G | -0.014 | 0.002 | 2.20E-10 |  | 0.017 | 0.016 | 0.281 |
| rs287104 | 19 | 34290995 | A | G | 0.012 | 0.002 | 4.40E-11 |  | 0.022 | 0.014 | 0.107 |
| rs2875762 | 6 | 124925032 | C | G | 0.014 | 0.002 | 1.20E-11 |  | -0.012 | 0.022 | 0.589 |
| rs2907948 | 7 | 150638484 | A | G | -0.014 | 0.002 | 1.30E-13 |  | -0.003 | 0.018 | 0.869 |
| rs2931434 | 5 | 73159098 | T | C | -0.010 | 0.002 | 1.40E-08 |  | 0.017 | 0.015 | 0.246 |
| rs2943465 | 12 | 19265921 | C | T | 0.025 | 0.004 | 2.00E-10 |  | 0.020 | 0.025 | 0.439 |
| rs294704 | 5 | 152519088 | T | G | -0.011 | 0.002 | 4.00E-09 |  | -0.005 | 0.016 | 0.763 |
| rs3007105 | 14 | 47367616 | T | C | 0.014 | 0.002 | 1.10E-17 |  | 0.002 | 0.014 | 0.912 |
| rs326896 | 4 | 112669571 | T | C | -0.013 | 0.002 | 2.80E-13 |  | 0.005 | 0.014 | 0.717 |
| rs331966 | 4 | 143675717 | C | A | 0.011 | 0.002 | 3.20E-10 |  | -0.007 | 0.014 | 0.607 |
| rs33500 | 3 | 42427191 | T | C | -0.017 | 0.002 | 4.30E-14 |  | 0.013 | 0.017 | 0.465 |
| rs339991 | 15 | 60913637 | G | A | 0.012 | 0.002 | 1.20E-12 |  | -0.001 | 0.014 | 0.934 |
| rs349088 | 11 | 84814393 | A | C | -0.013 | 0.002 | 1.80E-13 |  | -0.031 | 0.014 | 0.026 |
| rs3731695 | 2 | 203820275 | C | T | 0.012 | 0.002 | 7.90E-13 |  | 0.001 | 0.014 | 0.971 |
| rs3732084 | 2 | 207174316 | C | T | 0.011 | 0.002 | 1.10E-09 |  | -0.020 | 0.014 | 0.137 |
| rs3736485 | 15 | 51748610 | G | A | -0.013 | 0.002 | 2.50E-16 |  | 0.007 | 0.014 | 0.635 |
| rs3749897 | 6 | 42532102 | T | C | 0.012 | 0.002 | 8.40E-12 |  | 0.010 | 0.014 | 0.467 |
| rs3754963 | 2 | 166185707 | T | A | -0.012 | 0.002 | 3.30E-10 |  | 0.004 | 0.015 | 0.784 |
| rs3764835 | 2 | 159519368 | A | G | -0.014 | 0.002 | 3.10E-09 |  | 0.027 | 0.017 | 0.120 |
| rs3772882 | 3 | 81808602 | A | C | 0.013 | 0.002 | 6.60E-13 |  | -0.011 | 0.014 | 0.403 |
| rs3800229 | 6 | 108996963 | T | G | 0.018 | 0.002 | 1.40E-22 |  | -0.004 | 0.014 | 0.767 |
| rs3800637 | 7 | 137403432 | C | T | 0.012 | 0.002 | 5.10E-10 |  | -0.001 | 0.014 | 0.929 |
| rs3806114 | 6 | 20482335 | A | G | -0.011 | 0.002 | 3.40E-10 |  | -0.015 | 0.016 | 0.353 |
| rs3806572 | 2 | 55238677 | A | G | -0.015 | 0.002 | 1.60E-14 |  | 0.001 | 0.016 | 0.936 |
| rs3807645 | 7 | 77830091 | A | G | -0.017 | 0.002 | 2.40E-15 |  | -0.004 | 0.017 | 0.805 |
| rs380857 | 9 | 101491066 | A | C | -0.015 | 0.003 | 3.60E-08 |  | -0.018 | 0.018 | 0.302 |
| rs3814883 | 16 | 29994922 | T | C | 0.023 | 0.002 | 1.10E-40 |  | 0.005 | 0.014 | 0.699 |
| rs3828783 | 6 | 33767727 | A | G | -0.017 | 0.002 | 5.60E-15 |  | 0.007 | 0.016 | 0.647 |
| rs3829849 | 9 | 129390800 | T | C | 0.010 | 0.002 | 5.90E-09 |  | -0.004 | 0.014 | 0.760 |
| rs38314 | 7 | 70067315 | A | G | -0.012 | 0.002 | 4.70E-12 |  | -0.014 | 0.014 | 0.303 |
| rs3844598 | 5 | 140992235 | G | A | 0.010 | 0.002 | 3.80E-08 |  | -0.005 | 0.014 | 0.740 |
| rs3902951 | 14 | 69789755 | G | T | 0.013 | 0.002 | 7.00E-12 |  | 0.028 | 0.014 | 0.051 |
| rs3904244 | 10 | 27361527 | A | T | 0.016 | 0.003 | 4.30E-10 |  | 0.006 | 0.016 | 0.694 |
| rs391300 | 17 | 2216258 | C | T | -0.012 | 0.002 | 3.10E-12 |  | 0.000 | 0.014 | 0.995 |
| rs3935648 | 17 | 79085335 | G | C | -0.013 | 0.002 | 6.80E-09 |  | -0.004 | 0.018 | 0.842 |
| rs3977755 | 10 | 104420210 | T | C | -0.014 | 0.002 | 5.90E-13 |  | 0.026 | 0.017 | 0.125 |
| rs40067 | 5 | 107439012 | A | G | -0.027 | 0.002 | 7.10E-30 |  | 0.005 | 0.017 | 0.773 |
| rs4012234 | 20 | 32553047 | G | T | 0.014 | 0.002 | 9.90E-16 |  | 0.005 | 0.014 | 0.737 |
| rs4072917 | 8 | 143300279 | A | G | 0.012 | 0.002 | 6.90E-11 |  | 0.018 | 0.014 | 0.178 |
| rs4148155 | 4 | 89054667 | G | A | -0.019 | 0.003 | 5.00E-13 |  | -0.024 | 0.026 | 0.360 |
| rs4148866 | 12 | 123425575 | T | C | 0.010 | 0.002 | 4.00E-08 |  | 0.031 | 0.014 | 0.027 |
| rs4237643 | 11 | 43648368 | G | T | -0.022 | 0.002 | 4.30E-33 |  | -0.012 | 0.015 | 0.406 |
| rs427943 | 21 | 46570896 | C | A | 0.017 | 0.002 | 7.30E-23 |  | 0.010 | 0.014 | 0.451 |
| rs429343 | 2 | 147903382 | G | A | -0.015 | 0.002 | 6.80E-18 |  | -0.024 | 0.014 | 0.076 |
| rs4307239 | 7 | 24354300 | G | A | 0.012 | 0.002 | 3.90E-11 |  | -0.017 | 0.014 | 0.222 |
| rs4358081 | 2 | 29100642 | C | A | 0.010 | 0.002 | 1.50E-08 |  | -0.023 | 0.014 | 0.097 |
| rs4414033 | 1 | 156406853 | A | G | 0.013 | 0.002 | 1.40E-12 |  | 0.006 | 0.014 | 0.666 |
| rs4430672 | 14 | 63094407 | C | T | -0.013 | 0.002 | 3.90E-09 |  | -0.009 | 0.017 | 0.602 |
| rs4482463 | 2 | 205375909 | A | C | -0.033 | 0.003 | 2.80E-23 |  | 0.045 | 0.029 | 0.118 |
| rs4516268 | 17 | 1846831 | A | C | -0.022 | 0.002 | 5.20E-25 |  | -0.018 | 0.016 | 0.242 |
| rs4518345 | 5 | 27185904 | A | G | -0.012 | 0.002 | 1.00E-09 |  | -0.019 | 0.016 | 0.222 |
| rs4556997 | 2 | 100814858 | A | C | 0.020 | 0.002 | 6.90E-17 |  | -0.021 | 0.020 | 0.277 |
| rs4589691 | 2 | 144051398 | G | C | 0.014 | 0.002 | 4.70E-09 |  | 0.008 | 0.019 | 0.670 |
| rs4639527 | 2 | 416815 | G | A | 0.017 | 0.002 | 3.30E-20 |  | 0.015 | 0.014 | 0.277 |
| rs4653017 | 1 | 33776728 | T | C | 0.012 | 0.002 | 4.50E-11 |  | -0.005 | 0.014 | 0.712 |
| rs4660443 | 1 | 39591779 | T | C | 0.016 | 0.002 | 6.80E-15 |  | 0.028 | 0.017 | 0.100 |
| rs4722398 | 7 | 3125220 | T | C | 0.016 | 0.003 | 3.60E-10 |  | 0.009 | 0.027 | 0.743 |
| rs4740619 | 9 | 15634326 | C | T | -0.019 | 0.002 | 2.30E-30 |  | -0.004 | 0.014 | 0.789 |
| rs4757144 | 11 | 13331226 | A | G | 0.017 | 0.002 | 5.60E-22 |  | 0.010 | 0.014 | 0.450 |
| rs4783830 | 16 | 54255346 | A | G | -0.011 | 0.002 | 2.40E-08 |  | -0.003 | 0.015 | 0.858 |
| rs4786903 | 16 | 6697104 | G | A | 0.013 | 0.002 | 3.50E-10 |  | 0.018 | 0.016 | 0.246 |
| rs4800191 | 18 | 22461398 | C | G | 0.010 | 0.002 | 2.50E-09 |  | -0.015 | 0.014 | 0.293 |
| rs4813619 | 20 | 2815715 | T | G | -0.011 | 0.002 | 2.30E-09 |  | 0.019 | 0.014 | 0.169 |
| rs4818225 | 21 | 42629895 | G | A | 0.012 | 0.002 | 2.30E-10 |  | 0.026 | 0.014 | 0.059 |
| rs4820408 | 22 | 40604945 | G | T | -0.015 | 0.002 | 2.10E-19 |  | -0.031 | 0.014 | 0.023 |
| rs4842491 | 12 | 89905537 | T | C | 0.010 | 0.002 | 4.00E-08 |  | 0.013 | 0.015 | 0.387 |
| rs4851029 | 2 | 104159785 | G | T | 0.012 | 0.002 | 1.70E-12 |  | 0.009 | 0.014 | 0.495 |
| rs4858193 | 3 | 20441050 | C | T | -0.013 | 0.002 | 1.60E-11 |  | -0.032 | 0.015 | 0.041 |
| rs4864201 | 4 | 130731284 | C | T | -0.014 | 0.002 | 1.50E-16 |  | -0.012 | 0.014 | 0.387 |
| rs4880341 | 10 | 133992689 | T | C | -0.012 | 0.002 | 1.10E-11 |  | 0.003 | 0.014 | 0.843 |
| rs4906908 | 15 | 27040082 | G | T | 0.010 | 0.002 | 2.50E-09 |  | -0.002 | 0.014 | 0.861 |
| rs491711 | 11 | 28742220 | C | A | -0.012 | 0.002 | 1.10E-09 |  | 0.010 | 0.016 | 0.530 |
| rs4929923 | 11 | 8639200 | C | T | 0.018 | 0.002 | 7.20E-27 |  | 0.004 | 0.014 | 0.780 |
| rs4936175 | 11 | 132641959 | C | T | 0.012 | 0.002 | 1.40E-12 |  | 0.024 | 0.014 | 0.087 |
| rs4937870 | 11 | 112826709 | G | A | -0.011 | 0.002 | 8.80E-09 |  | 0.002 | 0.014 | 0.873 |
| rs4952843 | 2 | 46957845 | G | A | -0.013 | 0.002 | 6.80E-14 |  | 0.007 | 0.014 | 0.634 |
| rs4954638 | 2 | 137435455 | C | A | -0.012 | 0.002 | 2.90E-09 |  | -0.014 | 0.014 | 0.338 |
| rs4968656 | 17 | 61616959 | G | A | 0.012 | 0.002 | 8.20E-10 |  | 0.018 | 0.015 | 0.219 |
| rs4981693 | 14 | 29680331 | A | G | 0.021 | 0.002 | 6.90E-24 |  | -0.023 | 0.014 | 0.108 |
| rs4986044 | 17 | 21261560 | T | C | -0.016 | 0.002 | 3.30E-23 |  | -0.010 | 0.014 | 0.450 |
| rs538579 | 3 | 62711674 | C | G | 0.014 | 0.002 | 1.30E-13 |  | -0.027 | 0.015 | 0.081 |
| rs543874 | 1 | 177889480 | G | A | 0.048 | 0.002 | 1.20E-122 |  | 0.013 | 0.018 | 0.448 |
| rs559231 | 18 | 39644247 | T | G | 0.014 | 0.002 | 2.40E-14 |  | 0.019 | 0.014 | 0.171 |
| rs577525 | 10 | 99769388 | C | T | 0.017 | 0.002 | 9.70E-22 |  | 0.007 | 0.014 | 0.589 |
| rs592483 | 11 | 69445173 | T | C | -0.015 | 0.002 | 2.00E-18 |  | 0.028 | 0.014 | 0.040 |
| rs6050446 | 20 | 25195509 | G | A | 0.034 | 0.005 | 4.40E-13 |  | 0.108 | 0.045 | 0.016 |
| rs6235 | 5 | 95728898 | G | C | 0.018 | 0.002 | 1.50E-19 |  | 0.012 | 0.015 | 0.439 |
| rs6265 | 11 | 27679916 | T | C | -0.041 | 0.002 | 1.00E-86 |  | -0.033 | 0.019 | 0.077 |
| rs6443750 | 3 | 181329682 | C | T | 0.015 | 0.002 | 3.20E-12 |  | -0.006 | 0.017 | 0.709 |
| rs6448587 | 4 | 28561990 | C | A | -0.017 | 0.002 | 2.30E-13 |  | -0.033 | 0.016 | 0.039 |
| rs645040 | 3 | 135926622 | T | G | 0.017 | 0.002 | 2.50E-18 |  | 0.047 | 0.019 | 0.014 |
| rs6461115 | 7 | 2103668 | G | A | -0.014 | 0.002 | 1.20E-13 |  | 0.008 | 0.015 | 0.582 |
| rs6471941 | 8 | 62117973 | A | G | 0.016 | 0.002 | 3.10E-13 |  | 0.015 | 0.014 | 0.284 |
| rs6500208 | 16 | 49011249 | A | G | 0.014 | 0.002 | 4.10E-12 |  | 0.006 | 0.015 | 0.689 |
| rs6512302 | 20 | 62691550 | C | G | 0.014 | 0.002 | 2.10E-12 |  | -0.001 | 0.017 | 0.947 |
| rs6545714 | 2 | 59307725 | A | G | -0.019 | 0.002 | 9.10E-31 |  | 0.002 | 0.014 | 0.884 |
| rs6556301 | 5 | 176527577 | T | G | -0.011 | 0.002 | 4.10E-10 |  | 0.014 | 0.014 | 0.325 |
| rs6561943 | 13 | 58356761 | T | C | 0.012 | 0.002 | 4.20E-10 |  | 0.019 | 0.015 | 0.224 |
| rs657452 | 1 | 49589847 | G | A | -0.019 | 0.002 | 7.20E-29 |  | -0.006 | 0.014 | 0.668 |
| rs6587552 | 1 | 151018861 | G | A | -0.017 | 0.002 | 1.60E-17 |  | -0.015 | 0.015 | 0.312 |
| rs6591407 | 11 | 56914157 | A | C | -0.012 | 0.002 | 1.90E-08 |  | -0.010 | 0.020 | 0.628 |
| rs6593688 | 1 | 96322205 | G | A | 0.014 | 0.002 | 8.60E-15 |  | -0.001 | 0.014 | 0.934 |
| rs663129 | 18 | 57838401 | A | G | 0.055 | 0.002 | 1.60E-178 |  | 0.008 | 0.018 | 0.655 |
| rs6673081 | 1 | 154989595 | C | T | -0.01 | 0.002 | 1.80E-08 |  | -0.019 | 0.014 | 0.180 |
| rs6692586 | 1 | 23299906 | G | A | -0.019 | 0.002 | 1.10E-16 |  | 0.007 | 0.021 | 0.758 |
| rs6712 | 22 | 50637922 | C | G | 0.014 | 0.003 | 4.40E-08 |  | -0.021 | 0.020 | 0.286 |
| rs6764533 | 3 | 196088464 | A | G | 0.012 | 0.002 | 1.40E-10 |  | 0.011 | 0.015 | 0.477 |
| rs6772756 | 3 | 182312152 | G | A | -0.010 | 0.002 | 4.00E-08 |  | 0.001 | 0.014 | 0.931 |
| rs6785245 | 3 | 82647990 | C | T | 0.013 | 0.002 | 4.00E-14 |  | -0.007 | 0.014 | 0.625 |
| rs6804842 | 3 | 25106437 | G | A | 0.016 | 0.002 | 3.60E-21 |  | 0.010 | 0.014 | 0.449 |
| rs6841761 | 4 | 25423538 | T | G | -0.013 | 0.002 | 6.40E-16 |  | 0.003 | 0.014 | 0.817 |
| rs685870 | 11 | 64111928 | C | T | 0.012 | 0.002 | 2.40E-10 |  | -0.001 | 0.015 | 0.941 |
| rs6985109 | 8 | 10761585 | A | G | -0.018 | 0.002 | 1.50E-26 |  | -0.024 | 0.022 | 0.269 |
| rs7024334 | 9 | 109072075 | G | T | -0.014 | 0.002 | 3.10E-12 |  | 0.005 | 0.017 | 0.770 |
| rs7025938 | 9 | 103088321 | G | C | 0.017 | 0.002 | 3.70E-19 |  | 0.002 | 0.014 | 0.860 |
| rs7037266 | 9 | 6942940 | A | C | -0.011 | 0.002 | 3.50E-10 |  | -0.004 | 0.014 | 0.792 |
| rs705217 | 1 | 34581472 | G | T | -0.010 | 0.002 | 9.30E-09 |  | -0.002 | 0.014 | 0.911 |
| rs705704 | 12 | 56435412 | A | G | -0.013 | 0.002 | 1.90E-13 |  | -0.016 | 0.015 | 0.272 |
| rs7084454 | 10 | 21821274 | A | G | 0.019 | 0.002 | 4.00E-25 |  | 0.053 | 0.015 | 0.000 |
| rs709400 | 14 | 104149475 | G | A | -0.015 | 0.002 | 4.60E-19 |  | -0.009 | 0.015 | 0.544 |
| rs7102454 | 11 | 65594820 | C | T | 0.016 | 0.002 | 2.40E-18 |  | 0.008 | 0.015 | 0.612 |
| rs7117238 | 11 | 78040259 | A | G | -0.013 | 0.002 | 2.50E-09 |  | -0.005 | 0.017 | 0.761 |
| rs7124681 | 11 | 47529947 | A | C | 0.026 | 0.002 | 3.20E-58 |  | -0.008 | 0.014 | 0.589 |
| rs7138803 | 12 | 50247468 | A | G | 0.03 | 0.002 | 2.30E-71 |  | 0.001 | 0.014 | 0.927 |
| rs7144011 | 14 | 79940383 | T | G | 0.028 | 0.002 | 5.20E-47 |  | 0.008 | 0.016 | 0.602 |
| rs7148846 | 14 | 40133821 | G | T | 0.012 | 0.002 | 2.20E-08 |  | 0.038 | 0.016 | 0.016 |
| rs7172627 | 15 | 31877690 | G | A | 0.012 | 0.002 | 1.10E-11 |  | 0.012 | 0.014 | 0.385 |
| rs7181498 | 15 | 95271404 | C | T | -0.016 | 0.002 | 1.00E-19 |  | 0.011 | 0.014 | 0.420 |
| rs7196720 | 16 | 24534662 | C | T | -0.013 | 0.002 | 7.30E-14 |  | 0.006 | 0.014 | 0.638 |
| rs7206608 | 16 | 82872628 | G | C | 0.013 | 0.002 | 1.30E-12 |  | 0.041 | 0.014 | 0.004 |
| rs7222349 | 17 | 42304644 | A | G | 0.012 | 0.002 | 3.30E-10 |  | 0.005 | 0.015 | 0.734 |
| rs7239575 | 18 | 21120035 | C | T | -0.020 | 0.002 | 7.40E-32 |  | -0.018 | 0.014 | 0.189 |
| rs7318817 | 13 | 28617708 | T | C | -0.016 | 0.002 | 2.70E-18 |  | -0.020 | 0.014 | 0.155 |
| rs7334078 | 13 | 99120484 | C | T | -0.012 | 0.002 | 2.20E-10 |  | -0.003 | 0.016 | 0.842 |
| rs7358465 | 11 | 89990280 | T | C | 0.010 | 0.002 | 3.00E-08 |  | -0.011 | 0.015 | 0.453 |
| rs7488867 | 12 | 103699685 | T | C | -0.020 | 0.002 | 8.40E-24 |  | 0.007 | 0.015 | 0.651 |
| rs7498665 | 16 | 28883241 | G | A | 0.027 | 0.002 | 5.60E-60 |  | -0.007 | 0.014 | 0.618 |
| rs7535528 | 1 | 2444414 | A | G | -0.015 | 0.002 | 1.40E-16 |  | 0.003 | 0.014 | 0.842 |
| rs754635 | 3 | 42305131 | G | C | 0.020 | 0.003 | 2.20E-13 |  | 0.001 | 0.023 | 0.977 |
| rs7550711 | 1 | 110082886 | T | C | 0.065 | 0.005 | 3.20E-38 |  | -0.031 | 0.029 | 0.281 |
| rs7551507 | 1 | 74995225 | T | C | -0.018 | 0.002 | 9.30E-30 |  | -0.016 | 0.014 | 0.223 |
| rs7557796 | 2 | 86766153 | C | T | -0.016 | 0.002 | 2.30E-19 |  | 0.003 | 0.014 | 0.857 |
| rs756717 | 16 | 72996162 | A | G | -0.014 | 0.002 | 5.40E-18 |  | 0.010 | 0.014 | 0.483 |
| rs7599312 | 2 | 213413231 | A | G | -0.019 | 0.002 | 6.90E-24 |  | 0.026 | 0.015 | 0.083 |
| rs7615297 | 3 | 156299313 | G | C | -0.015 | 0.002 | 5.70E-10 |  | -0.009 | 0.018 | 0.612 |
| rs7626079 | 3 | 66427259 | T | C | 0.011 | 0.002 | 1.60E-09 |  | 0.026 | 0.015 | 0.073 |
| rs7637852 | 3 | 44041777 | G | A | -0.014 | 0.002 | 1.70E-13 |  | 0.004 | 0.014 | 0.798 |
| rs7640424 | 3 | 107820063 | T | C | -0.014 | 0.002 | 2.30E-14 |  | -0.012 | 0.014 | 0.412 |
| rs765875 | 6 | 143185683 | T | C | -0.012 | 0.002 | 3.00E-12 |  | -0.006 | 0.014 | 0.662 |
| rs7683836 | 4 | 180167906 | A | G | -0.011 | 0.002 | 6.30E-11 |  | -0.031 | 0.014 | 0.022 |
| rs7685048 | 4 | 95027784 | T | C | -0.010 | 0.002 | 4.10E-09 |  | 0.010 | 0.014 | 0.475 |
| rs768840 | 14 | 73143457 | A | G | 0.011 | 0.002 | 2.00E-10 |  | -0.014 | 0.014 | 0.322 |
| rs769449 | 19 | 45410002 | A | G | -0.025 | 0.003 | 2.30E-20 |  | -0.049 | 0.019 | 0.008 |
| rs7694732 | 4 | 115124089 | G | A | -0.010 | 0.002 | 8.70E-09 |  | 0.012 | 0.014 | 0.396 |
| rs7703576 | 5 | 144543996 | C | T | 0.010 | 0.002 | 4.80E-08 |  | 0.002 | 0.014 | 0.871 |
| rs7704281 | 5 | 50591460 | A | G | 0.027 | 0.004 | 6.50E-11 |  | 0.059 | 0.035 | 0.094 |
| rs7715256 | 5 | 153537893 | T | G | -0.017 | 0.002 | 2.20E-24 |  | 0.010 | 0.014 | 0.452 |
| rs7724675 | 5 | 130440010 | A | G | -0.012 | 0.002 | 9.50E-09 |  | 0.005 | 0.015 | 0.758 |
| rs7730004 | 5 | 43191033 | T | C | 0.015 | 0.002 | 9.10E-16 |  | -0.023 | 0.014 | 0.092 |
| rs7730898 | 5 | 170459675 | A | G | 0.017 | 0.002 | 4.50E-20 |  | 0.008 | 0.014 | 0.587 |
| rs774246 | 7 | 26990816 | G | A | 0.015 | 0.003 | 5.40E-10 |  | -0.045 | 0.021 | 0.031 |
| rs7761673 | 6 | 70357368 | A | T | -0.013 | 0.002 | 1.90E-09 |  | 0.006 | 0.019 | 0.769 |
| rs7780752 | 7 | 93241640 | C | T | 0.014 | 0.002 | 1.00E-14 |  | 0.003 | 0.015 | 0.846 |
| rs7788008 | 7 | 112972483 | A | G | -0.016 | 0.002 | 1.10E-19 |  | -0.034 | 0.014 | 0.014 |
| rs7811342 | 7 | 138794618 | C | T | -0.020 | 0.003 | 1.10E-11 |  | 0.006 | 0.018 | 0.749 |
| rs7819514 | 8 | 93204442 | A | G | -0.011 | 0.002 | 5.70E-09 |  | -0.006 | 0.014 | 0.648 |
| rs7826312 | 8 | 32400115 | C | T | 0.010 | 0.002 | 4.90E-10 |  | -0.010 | 0.014 | 0.449 |
| rs7844647 | 8 | 34503776 | C | T | -0.012 | 0.002 | 2.80E-11 |  | -0.039 | 0.016 | 0.013 |
| rs7869771 | 9 | 94180627 | C | A | -0.014 | 0.002 | 4.90E-13 |  | -0.013 | 0.017 | 0.444 |
| rs7871866 | 9 | 131027982 | C | G | 0.019 | 0.002 | 2.30E-14 |  | -0.018 | 0.017 | 0.308 |
| rs7899106 | 10 | 87410904 | G | A | 0.033 | 0.004 | 1.00E-18 |  | 0.010 | 0.033 | 0.755 |
| rs7903146 | 10 | 114758349 | T | C | -0.018 | 0.002 | 1.30E-23 |  | 0.015 | 0.017 | 0.392 |
| rs7925214 | 11 | 130794253 | T | C | 0.015 | 0.002 | 4.40E-17 |  | -0.001 | 0.014 | 0.950 |
| rs7970953 | 12 | 24075508 | A | G | 0.014 | 0.002 | 9.80E-14 |  | 0.011 | 0.015 | 0.441 |
| rs7983065 | 13 | 33380786 | T | C | -0.015 | 0.002 | 8.90E-18 |  | 0.011 | 0.014 | 0.430 |
| rs7998796 | 13 | 81020036 | G | A | 0.011 | 0.002 | 1.10E-08 |  | 0.005 | 0.014 | 0.750 |
| rs8036040 | 15 | 36402716 | A | C | 0.011 | 0.002 | 2.70E-10 |  | 0.007 | 0.014 | 0.599 |
| rs8047395 | 16 | 53798523 | A | G | 0.064 | 0.002 | 1.00E-200 |  | 0.000 | 0.014 | 0.985 |
| rs806600 | 5 | 172914939 | G | A | -0.010 | 0.002 | 3.30E-08 |  | -0.028 | 0.014 | 0.040 |
| rs8071182 | 17 | 55336155 | A | G | 0.013 | 0.002 | 2.10E-09 |  | -0.032 | 0.019 | 0.087 |
| rs8090983 | 18 | 52586691 | G | A | 0.012 | 0.002 | 2.00E-10 |  | 0.020 | 0.014 | 0.166 |
| rs8097672 | 18 | 1839601 | T | A | 0.02 | 0.003 | 8.40E-16 |  | 0.032 | 0.018 | 0.073 |
| rs8097783 | 18 | 58051294 | A | G | -0.039 | 0.003 | 7.20E-36 |  | -0.024 | 0.034 | 0.483 |
| rs8123881 | 20 | 15819495 | G | A | 0.020 | 0.002 | 4.40E-16 |  | 0.004 | 0.016 | 0.784 |
| rs8181823 | 13 | 65477940 | C | A | 0.013 | 0.002 | 4.10E-10 |  | -0.003 | 0.015 | 0.858 |
| rs818524 | 1 | 85201228 | C | T | 0.011 | 0.002 | 3.40E-08 |  | 0.006 | 0.015 | 0.687 |
| rs8192675 | 3 | 170724883 | C | T | 0.015 | 0.002 | 1.40E-17 |  | -0.006 | 0.015 | 0.689 |
| rs825688 | 16 | 73595718 | T | C | -0.010 | 0.002 | 4.70E-08 |  | 0.001 | 0.014 | 0.959 |
| rs845084 | 10 | 125220036 | A | G | 0.014 | 0.002 | 1.30E-12 |  | -0.004 | 0.016 | 0.795 |
| rs852056 | 20 | 17102860 | C | T | -0.013 | 0.002 | 1.80E-10 |  | 0.014 | 0.016 | 0.370 |
| rs865809 | 3 | 183997735 | G | A | -0.013 | 0.002 | 5.40E-10 |  | -0.005 | 0.015 | 0.740 |
| rs872281 | 14 | 40834177 | T | C | -0.015 | 0.002 | 4.70E-11 |  | 0.030 | 0.016 | 0.058 |
| rs876605 | 5 | 77801359 | G | A | -0.011 | 0.002 | 3.40E-08 |  | -0.026 | 0.016 | 0.094 |
| rs879620 | 16 | 4015729 | T | C | 0.023 | 0.002 | 5.30E-38 |  | -0.013 | 0.014 | 0.355 |
| rs889398 | 16 | 69556715 | T | C | -0.020 | 0.002 | 1.30E-32 |  | -0.014 | 0.014 | 0.318 |
| rs895330 | 19 | 4060707 | G | C | -0.020 | 0.002 | 5.50E-19 |  | -0.005 | 0.018 | 0.787 |
| rs901630 | 6 | 98539519 | T | C | -0.015 | 0.002 | 1.90E-18 |  | -0.003 | 0.014 | 0.822 |
| rs902695 | 2 | 113955074 | A | G | -0.010 | 0.002 | 2.20E-09 |  | 0.043 | 0.014 | 0.002 |
| rs9294260 | 6 | 83433228 | A | G | 0.015 | 0.002 | 1.80E-19 |  | 0.013 | 0.014 | 0.342 |
| rs9300422 | 13 | 98223320 | G | A | -0.010 | 0.002 | 4.00E-09 |  | -0.007 | 0.015 | 0.634 |
| rs930295 | 2 | 50233352 | C | A | -0.021 | 0.002 | 1.00E-19 |  | 0.024 | 0.020 | 0.235 |
| rs9304665 | 19 | 47602577 | A | T | 0.023 | 0.002 | 2.90E-29 |  | -0.001 | 0.015 | 0.969 |
| rs934224 | 2 | 16613889 | T | C | 0.011 | 0.002 | 4.70E-08 |  | 0.020 | 0.015 | 0.175 |
| rs9362662 | 6 | 90296588 | G | A | -0.011 | 0.002 | 1.20E-10 |  | -0.001 | 0.014 | 0.919 |
| rs9367368 | 6 | 13189275 | C | T | -0.012 | 0.002 | 1.00E-11 |  | 0.003 | 0.014 | 0.831 |
| rs9375702 | 6 | 130384187 | T | C | -0.012 | 0.002 | 7.90E-10 |  | 0.024 | 0.014 | 0.090 |
| rs9379827 | 6 | 26153335 | A | C | -0.013 | 0.002 | 6.90E-12 |  | -0.045 | 0.016 | 0.005 |
| rs9408882 | 9 | 118664402 | A | G | -0.009 | 0.002 | 1.30E-08 |  | 0.003 | 0.014 | 0.808 |
| rs946824 | 1 | 243684019 | C | T | -0.021 | 0.003 | 1.10E-15 |  | 0.006 | 0.019 | 0.772 |
| rs947612 | 6 | 73738661 | A | G | -0.012 | 0.002 | 5.60E-09 |  | -0.013 | 0.015 | 0.405 |
| rs9522285 | 13 | 112230701 | A | G | 0.013 | 0.002 | 2.50E-13 |  | 0.026 | 0.014 | 0.059 |
| rs9538162 | 13 | 59265043 | C | T | -0.016 | 0.002 | 4.80E-19 |  | -0.022 | 0.014 | 0.107 |
| rs9547153 | 13 | 85903717 | G | A | 0.010 | 0.002 | 8.70E-09 |  | 0.001 | 0.014 | 0.919 |
| rs9571687 | 13 | 67472713 | A | C | -0.013 | 0.002 | 2.80E-12 |  | -0.015 | 0.016 | 0.346 |
| rs9615905 | 22 | 48875699 | T | C | 0.011 | 0.002 | 2.70E-10 |  | 0.029 | 0.014 | 0.034 |
| rs962273 | 17 | 46978353 | C | T | 0.014 | 0.002 | 2.60E-13 |  | 0.022 | 0.015 | 0.151 |
| rs9650755 | 9 | 96484342 | G | A | 0.015 | 0.002 | 2.80E-15 |  | 0.008 | 0.015 | 0.591 |
| rs9688431 | 6 | 73922654 | C | T | -0.023 | 0.004 | 2.40E-11 |  | -0.033 | 0.040 | 0.411 |
| rs977747 | 1 | 47684677 | G | T | -0.017 | 0.002 | 1.30E-24 |  | 0.021 | 0.014 | 0.130 |
| rs9783858 | 18 | 42534584 | T | C | 0.009 | 0.002 | 3.30E-08 |  | 0.012 | 0.014 | 0.391 |
| rs9806742 | 15 | 73051219 | A | G | 0.021 | 0.003 | 1.40E-15 |  | 0.042 | 0.030 | 0.156 |
| rs9816226 | 3 | 185834499 | T | A | 0.032 | 0.002 | 1.60E-52 |  | 0.013 | 0.018 | 0.498 |
| rs9845966 | 3 | 13433158 | G | T | -0.011 | 0.002 | 2.50E-10 |  | 0.003 | 0.014 | 0.816 |
| rs987237 | 6 | 50803050 | G | A | 0.041 | 0.002 | 9.30E-84 |  | 0.010 | 0.017 | 0.530 |
| rs9926784 | 16 | 19941968 | C | T | -0.026 | 0.002 | 9.90E-35 |  | 0.002 | 0.018 | 0.923 |
| rs9927848 | 16 | 23833071 | A | C | -0.012 | 0.002 | 6.40E-10 |  | -0.018 | 0.017 | 0.303 |
| rs9951619 | 18 | 56882326 | G | T | 0.016 | 0.002 | 1.40E-15 |  | -0.006 | 0.014 | 0.664 |
| rs998732 | 19 | 19378671 | G | A | -0.017 | 0.002 | 2.00E-14 |  | 0.017 | 0.022 | 0.457 |
| rs9989141 | 14 | 94006257 | T | C | 0.016 | 0.002 | 3.60E-21 |  | 0.000 | 0.014 | 0.982 |
| rs999889 | 10 | 84279949 | A | G | -0.011 | 0.002 | 1.40E-08 |  | -0.015 | 0.016 | 0.331 |

EA, effect allele; OA, other allele; SNP, single nucleotide polymorphism; SE, standard error.
