# Supplementary material for: Dried fruit intake causally protects against low back pain: A Mendelian randomization study
Source: Front Nutr. 2023 Mar 23;10:1027481. doi: 10.3389/fnut.2023.1027481 (PMC10076586; doi:10.3389/fnut.2023.1027481)
Supplement: Supplementary file 6 [file Table_6.DOCX]

Supplementary Table S6 Characteristics of the instruments for current tobacco smoking and their associations with low back pain.

| **SNP** | **Chr** | **Position** | **EA** | **OA** | **Exposure effect** |  |  |  | **Outcome effect** |  |  |
| --- | --- | --- | --- | --- | --- | --- | --- | --- | --- | --- | --- |
|  |  |  |  |  | **β** | **SE** | ***P*** |  | **β** | **SE** | ***P*** |
| rs10134246 | 14 | 80356531 | A | G | 0.012 | 0.002 | 7.30E-09 |  | 0.014 | 0.025 | 0.584 |
| rs10786717 | 10 | 104625237 | G | A | 0.008 | 0.001 | 1.90E-10 |  | -0.022 | 0.014 | 0.130 |
| rs10891481 | 11 | 112830562 | G | A | 0.008 | 0.001 | 5.90E-13 |  | 0.022 | 0.014 | 0.101 |
| rs11096777 | 4 | 35568903 | C | T | 0.009 | 0.001 | 1.70E-10 |  | 0.009 | 0.017 | 0.615 |
| rs11596214 | 10 | 106453832 | A | G | -0.006 | 0.001 | 4.10E-08 |  | -0.002 | 0.014 | 0.862 |
| rs12469094 | 2 | 22967228 | C | A | -0.007 | 0.001 | 2.20E-08 |  | -0.016 | 0.014 | 0.259 |
| rs12666306 | 7 | 115082406 | G | A | -0.009 | 0.001 | 8.80E-16 |  | -0.040 | 0.014 | 0.004 |
| rs1452787 | 18 | 53207207 | G | A | 0.007 | 0.001 | 2.80E-08 |  | -0.008 | 0.015 | 0.598 |
| rs1549212 | 5 | 166996722 | T | C | -0.008 | 0.001 | 1.70E-10 |  | -0.012 | 0.014 | 0.390 |
| rs1565735 | 8 | 27426077 | A | T | -0.010 | 0.001 | 1.20E-12 |  | -0.027 | 0.019 | 0.159 |
| rs2047502 | 9 | 128178894 | A | C | 0.007 | 0.001 | 7.80E-10 |  | -0.009 | 0.014 | 0.526 |
| rs214904 | 11 | 17225436 | T | C | -0.007 | 0.001 | 1.60E-08 |  | -0.001 | 0.014 | 0.952 |
| rs2588978 | 10 | 63559609 | C | T | -0.007 | 0.001 | 1.00E-08 |  | -0.015 | 0.014 | 0.271 |
| rs2740776 | 8 | 92003130 | T | C | -0.007 | 0.001 | 1.90E-08 |  | -0.042 | 0.014 | 0.004 |
| rs28545614 | 2 | 105994827 | T | C | 0.009 | 0.002 | 1.30E-08 |  | 0.033 | 0.021 | 0.120 |
| rs3001723 | 1 | 44037685 | A | G | 0.007 | 0.001 | 5.70E-09 |  | 0.013 | 0.015 | 0.388 |
| rs3025316 | 9 | 136459543 | C | T | 0.020 | 0.002 | 5.00E-28 |  | 0.053 | 0.039 | 0.170 |
| rs3087898 | 2 | 61765074 | A | G | -0.006 | 0.001 | 2.60E-08 |  | -0.003 | 0.014 | 0.850 |
| rs329120 | 5 | 133861756 | T | C | -0.007 | 0.001 | 7.70E-09 |  | -0.001 | 0.014 | 0.932 |
| rs34488670 | 15 | 47684936 | C | T | 0.010 | 0.001 | 2.70E-12 |  | 0.040 | 0.016 | 0.014 |
| rs3742365 | 14 | 104198251 | C | T | 0.007 | 0.001 | 9.50E-09 |  | 0.017 | 0.014 | 0.225 |
| rs4543592 | 9 | 3014254 | C | T | 0.006 | 0.001 | 3.40E-08 |  | -0.008 | 0.014 | 0.585 |
| rs4809542 | 20 | 61986787 | G | C | 0.017 | 0.002 | 7.00E-14 |  | 0.007 | 0.021 | 0.759 |
| rs4955411 | 3 | 49145304 | G | A | -0.008 | 0.001 | 4.10E-08 |  | -0.049 | 0.017 | 0.004 |
| rs56113850 | 19 | 41353107 | C | T | -0.013 | 0.001 | 4.20E-28 |  | -0.003 | 0.014 | 0.821 |
| rs6727997 | 2 | 146346285 | G | A | 0.007 | 0.001 | 9.00E-10 |  | 0.012 | 0.015 | 0.406 |
| rs6951574 | 7 | 153491516 | C | T | 0.007 | 0.001 | 2.60E-09 |  | 0.013 | 0.014 | 0.341 |
| rs7155595 | 14 | 77502546 | C | A | 0.007 | 0.001 | 1.20E-08 |  | 0.013 | 0.015 | 0.366 |
| rs72804566 | 2 | 58009423 | T | A | 0.017 | 0.003 | 2.00E-08 |  | 0.023 | 0.032 | 0.468 |
| rs7569203 | 2 | 45154418 | C | A | 0.007 | 0.001 | 2.00E-08 |  | 0.003 | 0.014 | 0.835 |
| rs7689452 | 4 | 147945733 | G | A | -0.007 | 0.001 | 1.60E-09 |  | -0.015 | 0.014 | 0.297 |
| rs7807019 | 7 | 117543063 | G | A | 0.007 | 0.001 | 6.90E-11 |  | 0.026 | 0.014 | 0.061 |
| rs8031550 | 15 | 80956407 | A | G | -0.008 | 0.001 | 2.10E-09 |  | 0.026 | 0.016 | 0.102 |
| rs9607805 | 22 | 41854446 | T | C | 0.008 | 0.001 | 1.20E-10 |  | -0.010 | 0.014 | 0.503 |

EA, effect allele; OA, other allele; SNP, single nucleotide polymorphism; SE, standard error.
