# Supplementary material for: Dried fruit intake causally protects against low back pain: A Mendelian randomization study
Source: Front Nutr. 2023 Mar 23;10:1027481. doi: 10.3389/fnut.2023.1027481 (PMC10076586; doi:10.3389/fnut.2023.1027481)
Supplement: Supplementary file 7 [file Table_7.DOCX]

Supplementary Table S7 Characteristics of the instruments for alcohol intake frequency and their associations with low back pain.

| **SNP** | **Chr** | **Position** | **EA** | **OA** | **Exposure effect** |  |  |  | **Outcome effect** |  |  |
| --- | --- | --- | --- | --- | --- | --- | --- | --- | --- | --- | --- |
|  |  |  |  |  | **β** | **SE** | ***P*** |  | **β** | **SE** | ***P*** |
| rs10188314 | 2 | 215402926 | T | C | -0.020 | 0.003 | 7.20E-11 |  | -0.016 | 0.014 | 0.248 |
| rs10792669 | 11 | 82688356 | G | A | 0.017 | 0.003 | 9.90E-09 |  | 0.016 | 0.014 | 0.224 |
| rs11039429 | 11 | 47867059 | T | C | -0.024 | 0.003 | 8.70E-15 |  | 0.005 | 0.014 | 0.710 |
| rs11223617 | 11 | 133780757 | A | G | 0.025 | 0.004 | 2.30E-11 |  | -0.002 | 0.015 | 0.920 |
| rs11700855 | 21 | 34270051 | G | A | -0.030 | 0.005 | 1.20E-08 |  | 0.008 | 0.021 | 0.714 |
| rs11750777 | 5 | 166830787 | A | G | -0.020 | 0.004 | 3.80E-08 |  | 1.00E-04 | 0.019 | 0.996 |
| rs11787216 | 8 | 142615222 | T | C | 0.024 | 0.003 | 2.40E-14 |  | 0.023 | 0.014 | 0.096 |
| rs11940694 | 4 | 39414993 | G | A | -0.044 | 0.003 | 1.00E-44 |  | -0.011 | 0.014 | 0.457 |
| rs12153855 | 6 | 32074804 | C | T | 0.029 | 0.005 | 2.40E-09 |  | 0.073 | 0.029 | 0.011 |
| rs1228589 | 3 | 131634826 | A | G | 0.021 | 0.004 | 2.30E-09 |  | 0.028 | 0.016 | 0.075 |
| rs1229984 | 4 | 100239319 | C | T | -0.262 | 0.009 | 1.40E-178 |  | -0.05 | 0.094 | 0.596 |
| rs12312693 | 12 | 57511734 | C | T | -0.018 | 0.003 | 6.80E-09 |  | -0.008 | 0.014 | 0.564 |
| rs13102973 | 4 | 135900688 | C | T | -0.019 | 0.003 | 4.90E-10 |  | -0.003 | 0.014 | 0.831 |
| rs13135092 | 4 | 103198082 | G | A | 0.044 | 0.005 | 1.60E-15 |  | 0.120 | 0.052 | 0.022 |
| rs13178443 | 5 | 145615275 | T | C | -0.019 | 0.003 | 3.80E-08 |  | -0.025 | 0.015 | 0.083 |
| rs13390019 | 2 | 97797680 | C | T | 0.030 | 0.004 | 4.30E-11 |  | 0.025 | 0.028 | 0.380 |
| rs1421085 | 16 | 53800954 | C | T | 0.020 | 0.003 | 1.00E-10 |  | 0.020 | 0.014 | 0.141 |
| rs1515591 | 3 | 174213976 | G | T | 0.018 | 0.003 | 4.90E-09 |  | 0.003 | 0.014 | 0.830 |
| rs1666658 | 11 | 121801129 | C | T | 0.018 | 0.003 | 6.70E-09 |  | 2.00E-04 | 0.014 | 0.986 |
| rs17662759 | 2 | 193989223 | C | T | 0.030 | 0.005 | 3.40E-08 |  | 0.037 | 0.022 | 0.095 |
| rs17690703 | 17 | 43925297 | T | C | 0.025 | 0.003 | 2.90E-13 |  | 0.038 | 0.021 | 0.075 |
| rs186347 | 14 | 59072226 | T | G | 0.018 | 0.003 | 4.00E-09 |  | 0.004 | 0.014 | 0.760 |
| rs1937522 | 13 | 68080817 | G | A | 0.017 | 0.003 | 2.50E-08 |  | 0.016 | 0.014 | 0.244 |
| rs1991083 | 2 | 23887437 | T | C | -0.022 | 0.003 | 6.30E-12 |  | 0.013 | 0.016 | 0.418 |
| rs2043677 | 18 | 38328207 | T | C | 0.026 | 0.004 | 1.60E-09 |  | -0.024 | 0.019 | 0.213 |
| rs2159935 | 4 | 55521017 | A | G | -0.019 | 0.003 | 8.30E-10 |  | 0.001 | 0.014 | 0.940 |
| rs2160935 | 8 | 30840651 | T | C | -0.019 | 0.003 | 1.40E-09 |  | -0.002 | 0.014 | 0.873 |
| rs2244598 | 1 | 216681000 | C | T | -0.018 | 0.003 | 3.80E-09 |  | 0.019 | 0.014 | 0.168 |
| rs2411453 | 16 | 28632021 | G | T | -0.035 | 0.003 | 7.30E-30 |  | -0.009 | 0.014 | 0.497 |
| rs2535911 | 14 | 73523162 | T | C | -0.019 | 0.003 | 2.70E-09 |  | -0.002 | 0.014 | 0.873 |
| rs2622167 | 7 | 153486704 | A | G | -0.019 | 0.003 | 4.60E-10 |  | 0.005 | 0.015 | 0.715 |
| rs262240 | 3 | 68408109 | T | C | -0.017 | 0.003 | 1.40E-08 |  | 0.006 | 0.014 | 0.651 |
| rs2717063 | 2 | 58110969 | A | C | -0.020 | 0.003 | 4.00E-11 |  | 0.027 | 0.014 | 0.049 |
| rs28768122 | 12 | 123885974 | C | T | 0.021 | 0.004 | 5.60E-09 |  | 0.031 | 0.016 | 0.052 |
| rs28787109 | 1 | 51218695 | A | G | 0.018 | 0.003 | 7.70E-09 |  | 0.0237 | 0.016 | 0.134 |
| rs2924321 | 18 | 53125435 | A | G | -0.020 | 0.003 | 1.60E-10 |  | -0.001 | 0.014 | 0.929 |
| rs2977454 | 8 | 141539923 | G | C | -0.026 | 0.005 | 1.70E-08 |  | -0.024 | 0.018 | 0.179 |
| rs34440851 | 8 | 87214346 | T | C | -0.023 | 0.004 | 4.60E-08 |  | 0.004 | 0.016 | 0.788 |
| rs34473884 | 10 | 133761285 | A | G | -0.020 | 0.004 | 6.20E-09 |  | 0.008 | 0.016 | 0.600 |
| rs34631026 | 16 | 6172126 | T | C | -0.017 | 0.003 | 2.90E-08 |  | 0.015 | 0.014 | 0.281 |
| rs34811474 | 4 | 25408838 | A | G | -0.020 | 0.004 | 1.90E-08 |  | -0.007 | 0.016 | 0.656 |
| rs35105141 | 16 | 30057148 | T | C | 0.026 | 0.003 | 1.40E-17 |  | 0.002 | 0.014 | 0.897 |
| rs362307 | 4 | 3241845 | T | C | 0.043 | 0.006 | 8.40E-14 |  | 0.047 | 0.027 | 0.080 |
| rs4241258 | 2 | 74226102 | T | C | 0.025 | 0.004 | 1.30E-08 |  | 0.046 | 0.023 | 0.045 |
| rs4242715 | 10 | 133986135 | A | G | -0.019 | 0.003 | 9.30E-09 |  | -0.019 | 0.014 | 0.177 |
| rs4417025 | 1 | 35363679 | A | G | -0.019 | 0.003 | 2.70E-09 |  | -0.005 | 0.016 | 0.758 |
| rs4503294 | 1 | 940096 | T | C | 0.018 | 0.003 | 3.40E-09 |  | -0.006 | 0.014 | 0.668 |
| rs461599 | 5 | 144136931 | C | A | -0.019 | 0.003 | 2.70E-10 |  | 0.010 | 0.014 | 0.469 |
| rs4726481 | 7 | 141668403 | T | G | 0.022 | 0.003 | 2.30E-12 |  | -0.011 | 0.014 | 0.454 |
| rs473098 | 2 | 45139779 | T | C | -0.022 | 0.003 | 9.10E-13 |  | 0.016 | 0.014 | 0.255 |
| rs489062 | 10 | 99715744 | A | G | 0.017 | 0.003 | 4.90E-08 |  | 0.022 | 0.014 | 0.104 |
| rs4916723 | 5 | 87854395 | C | A | 0.024 | 0.003 | 1.10E-14 |  | 0.022 | 0.014 | 0.099 |
| rs4940926 | 18 | 57734857 | C | T | -0.019 | 0.003 | 2.80E-08 |  | 0.005 | 0.017 | 0.795 |
| rs4968391 | 17 | 57780943 | T | G | -0.019 | 0.003 | 2.30E-09 |  | -0.008 | 0.014 | 0.576 |
| rs5022348 | 18 | 22639237 | T | C | 0.020 | 0.004 | 1.40E-08 |  | 0.018 | 0.014 | 0.174 |
| rs550942 | 11 | 58394154 | T | C | 0.022 | 0.004 | 2.00E-08 |  | 0.013 | 0.023 | 0.577 |
| rs56194430 | 5 | 67824690 | T | C | 0.023 | 0.004 | 3.10E-08 |  | -0.0123 | 0.020 | 0.549 |
| rs58905411 | 12 | 54623132 | A | G | -0.027 | 0.003 | 5.10E-18 |  | -0.064 | 0.014 | 0.000 |
| rs6030200 | 20 | 35554361 | A | G | -0.020 | 0.003 | 2.40E-09 |  | 0.009 | 0.014 | 0.516 |
| rs61873510 | 10 | 102626510 | T | G | 0.020 | 0.003 | 6.90E-10 |  | 0.016 | 0.015 | 0.299 |
| rs62305780 | 4 | 100290815 | G | C | -0.049 | 0.005 | 9.90E-22 |  | 0.015 | 0.019 | 0.455 |
| rs62339673 | 4 | 184828533 | A | C | 0.018 | 0.003 | 6.60E-09 |  | 0.019 | 0.014 | 0.179 |
| rs62466318 | 7 | 73042085 | T | C | -0.025 | 0.004 | 1.40E-11 |  | -0.005 | 0.017 | 0.780 |
| rs650558 | 17 | 40721042 | T | C | 0.021 | 0.004 | 3.40E-09 |  | -5.00E-04 | 0.017 | 0.975 |
| rs6727281 | 2 | 65558588 | T | C | -0.024 | 0.004 | 5.50E-10 |  | -0.005 | 0.015 | 0.760 |
| rs6943160 | 7 | 99886509 | C | T | 0.021 | 0.004 | 3.10E-08 |  | 0.020 | 0.016 | 0.226 |
| rs71651683 | 22 | 24828853 | T | C | -0.070 | 0.013 | 3.60E-08 |  | -0.009 | 0.079 | 0.912 |
| rs72769229 | 2 | 2220795 | T | A | -0.023 | 0.004 | 3.40E-08 |  | -0.006 | 0.021 | 0.792 |
| rs72787062 | 16 | 72105844 | A | G | -0.028 | 0.004 | 6.40E-12 |  | 0.037 | 0.019 | 0.050 |
| rs728538 | 16 | 51205819 | G | T | 0.023 | 0.004 | 1.80E-08 |  | 0.008 | 0.017 | 0.665 |
| rs7298932 | 12 | 23727301 | G | A | -0.024 | 0.004 | 3.80E-08 |  | 0.006 | 0.021 | 0.760 |
| rs7302200 | 12 | 56449435 | A | G | -0.018 | 0.003 | 8.40E-09 |  | -0.019 | 0.015 | 0.200 |
| rs73050128 | 7 | 1961882 | A | C | -0.026 | 0.004 | 2.10E-10 |  | 2.00E-04 | 0.016 | 0.990 |
| rs7330939 | 13 | 49971400 | T | C | -0.021 | 0.003 | 3.70E-10 |  | 0.002 | 0.014 | 0.911 |
| rs74679146 | 9 | 16287769 | C | T | -0.032 | 0.006 | 2.50E-08 |  | -0.024 | 0.026 | 0.349 |
| rs7514579 | 1 | 94051350 | C | A | 0.020 | 0.004 | 4.60E-08 |  | -0.027 | 0.017 | 0.106 |
| rs76082653 | 3 | 49029468 | T | C | 0.046 | 0.007 | 3.80E-12 |  | 0.051 | 0.038 | 0.184 |
| rs7610856 | 3 | 71579022 | A | C | -0.024 | 0.003 | 7.70E-15 |  | 0.007 | 0.014 | 0.637 |
| rs780094 | 2 | 27741237 | C | T | -0.051 | 0.003 | 1.30E-60 |  | -0.009 | 0.014 | 0.533 |
| rs780569 | 1 | 4569436 | A | T | 0.020 | 0.003 | 4.00E-09 |  | 7.00E-04 | 0.016 | 0.964 |
| rs80292319 | 15 | 76508632 | C | T | -0.039 | 0.006 | 1.40E-09 |  | -0.041 | 0.025 | 0.094 |
| rs8043563 | 16 | 19982353 | C | G | 0.023 | 0.003 | 1.70E-11 |  | 0.003 | 0.015 | 0.836 |
| rs838145 | 19 | 49248730 | A | G | 0.022 | 0.003 | 6.70E-13 |  | 0.031 | 0.014 | 0.028 |
| rs8614 | 17 | 27588806 | A | C | 0.025 | 0.004 | 2.70E-10 |  | -0.01 | 0.019 | 0.597 |
| rs9349379 | 6 | 12903957 | G | A | -0.019 | 0.003 | 3.50E-10 |  | -0.025 | 0.014 | 0.065 |
| rs9372625 | 6 | 98344031 | A | G | -0.026 | 0.003 | 2.90E-16 |  | -0.011 | 0.015 | 0.442 |
| rs9403297 | 6 | 141705482 | A | G | 0.019 | 0.003 | 1.80E-09 |  | -0.003 | 0.015 | 0.841 |
| rs9648478 | 7 | 39325802 | A | G | 0.017 | 0.003 | 2.60E-08 |  | 8.00E-04 | 0.014 | 0.952 |
| rs9814516 | 3 | 85407980 | T | G | -0.025 | 0.004 | 1.60E-12 |  | 0.009 | 0.016 | 0.570 |
| rs9829192 | 3 | 38569463 | T | G | 0.017 | 0.003 | 2.80E-08 |  | -0.006 | 0.014 | 0.657 |
| rs9906502 | 17 | 7615745 | A | G | 0.024 | 0.004 | 1.90E-09 |  | -0.002 | 0.017 | 0.889 |

EA, effect allele; OA, other allele; SNP, single nucleotide polymorphism; SE, standard error.
