# Supplementary material for: Dried fruit intake causally protects against low back pain: A Mendelian randomization study
Source: Front Nutr. 2023 Mar 23;10:1027481. doi: 10.3389/fnut.2023.1027481 (PMC10076586; doi:10.3389/fnut.2023.1027481)
Supplement: Supplementary file 8 [file Table_8.DOCX]

Supplementary Table S8 Characteristics of the instruments for total body bone mineral density and their associations with low back pain.

| **SNP** | **Chr** | **Position** | **EA** | **OA** | **Exposure effect** |  |  |  | **Outcome effect** |  |  |
| --- | --- | --- | --- | --- | --- | --- | --- | --- | --- | --- | --- |
|  |  |  |  |  | **β** | **SE** | ***P*** |  | **β** | **SE** | ***P*** |
| rs10048745 | 2 | 68962137 | A | G | -0.039 | 0.007 | 6.44E-09 |  | 0.003 | 0.016 | 0.860 |
| rs1037011 | 12 | 107302778 | C | T | 0.040 | 0.006 | 1.54E-12 |  | -0.017 | 0.014 | 0.213 |
| rs10490046 | 2 | 40630678 | C | A | -0.043 | 0.007 | 1.43E-10 |  | 0.012 | 0.014 | 0.384 |
| rs10493013 | 1 | 22703035 | C | T | 0.101 | 0.007 | 4.07E-43 |  | -0.028 | 0.024 | 0.244 |
| rs10735851 | 12 | 53743064 | A | G | -0.054 | 0.006 | 5.84E-18 |  | 0.004 | 0.015 | 0.803 |
| rs10777212 | 12 | 90334829 | T | G | 0.045 | 0.006 | 5.05E-14 |  | 0.010 | 0.016 | 0.548 |
| rs10788264 | 10 | 124015986 | A | G | -0.034 | 0.006 | 2.61E-09 |  | -0.024 | 0.014 | 0.082 |
| rs10832520 | 11 | 15816918 | A | T | 0.112 | 0.016 | 1.00E-12 |  | 0.070 | 0.047 | 0.133 |
| rs10901216 | 9 | 133471891 | A | G | -0.047 | 0.006 | 5.53E-15 |  | -0.009 | 0.014 | 0.527 |
| rs10931982 | 2 | 202832130 | C | T | 0.051 | 0.009 | 1.59E-08 |  | 0.035 | 0.019 | 0.066 |
| rs11228240 | 11 | 68218290 | T | C | -0.083 | 0.007 | 1.72E-35 |  | 0.038 | 0.018 | 0.038 |
| rs1159798 | 10 | 54412493 | C | A | -0.043 | 0.007 | 1.01E-09 |  | 0.000 | 0.016 | 0.999 |
| rs11745493 | 5 | 122847622 | G | A | -0.045 | 0.007 | 7.74E-12 |  | 0.016 | 0.017 | 0.325 |
| rs117557198 | 12 | 49655948 | G | A | 0.077 | 0.012 | 1.58E-10 |  | -0.015 | 0.024 | 0.542 |
| rs118115924 | 12 | 49379537 | T | G | -0.282 | 0.030 | 6.99E-21 |  | -0.525 | 0.185 | 0.004 |
| rs11898505 | 2 | 54684557 | G | A | -0.034 | 0.006 | 1.28E-08 |  | 0.019 | 0.016 | 0.222 |
| rs11904127 | 2 | 85484818 | A | G | -0.032 | 0.006 | 1.18E-08 |  | 0.017 | 0.014 | 0.209 |
| rs11910328 | 21 | 40350744 | A | G | -0.043 | 0.008 | 2.99E-08 |  | 0.001 | 0.018 | 0.974 |
| rs11934731 | 4 | 88831249 | A | G | -0.067 | 0.006 | 8.39E-29 |  | 0.000 | 0.015 | 0.996 |
| rs12044944 | 1 | 240581653 | T | C | 0.055 | 0.007 | 7.54E-14 |  | -0.012 | 0.020 | 0.545 |
| rs12258451 | 10 | 54423853 | G | C | -0.070 | 0.009 | 2.41E-15 |  | -0.009 | 0.020 | 0.658 |
| rs12442242 | 15 | 38340874 | G | A | 0.051 | 0.008 | 4.94E-10 |  | 0.021 | 0.021 | 0.310 |
| rs12534510 | 7 | 120730944 | C | A | 0.040 | 0.006 | 3.15E-12 |  | 0.019 | 0.014 | 0.170 |
| rs12612325 | 2 | 119632252 | A | G | -0.055 | 0.008 | 1.98E-12 |  | 0.016 | 0.017 | 0.351 |
| rs1286150 | 14 | 91464890 | C | T | 0.055 | 0.007 | 2.44E-14 |  | 0.002 | 0.019 | 0.937 |
| rs13204965 | 6 | 127167072 | C | A | -0.062 | 0.007 | 1.02E-18 |  | 0.023 | 0.018 | 0.194 |
| rs143187557 | 11 | 47284279 | T | C | -0.124 | 0.020 | 1.15E-09 |  | -0.014 | 0.036 | 0.705 |
| rs144279715 | 2 | 119548256 | G | A | 0.230 | 0.029 | 6.18E-15 |  | -0.064 | 0.046 | 0.162 |
| rs144691710 | 17 | 41819562 | G | A | 0.102 | 0.011 | 2.24E-19 |  | -0.007 | 0.021 | 0.741 |
| rs1452102 | 21 | 28773868 | G | T | 0.035 | 0.006 | 1.74E-09 |  | 0.007 | 0.014 | 0.639 |
| rs1548607 | 7 | 50901491 | G | A | -0.036 | 0.007 | 4.18E-08 |  | 0.033 | 0.014 | 0.020 |
| rs2252865 | 1 | 8422676 | C | T | 0.033 | 0.006 | 4.72E-08 |  | 0.005 | 0.014 | 0.715 |
| rs2289410 | 2 | 42284110 | T | A | -0.049 | 0.009 | 2.00E-08 |  | -0.020 | 0.029 | 0.487 |
| rs2350085 | 2 | 202799604 | C | T | 0.064 | 0.009 | 3.79E-14 |  | 0.022 | 0.018 | 0.225 |
| rs2414098 | 15 | 51537806 | C | T | 0.033 | 0.006 | 1.99E-08 |  | -0.014 | 0.014 | 0.315 |
| rs2566751 | 1 | 68664913 | A | T | -0.057 | 0.010 | 1.32E-08 |  | -0.009 | 0.022 | 0.684 |
| rs2566752 | 1 | 68656697 | C | T | 0.072 | 0.006 | 1.88E-34 |  | -0.003 | 0.014 | 0.857 |
| rs2873195 | 17 | 2064702 | T | A | 0.041 | 0.006 | 4.31E-11 |  | 0.010 | 0.015 | 0.505 |
| rs34102936 | 7 | 38142840 | A | G | 0.047 | 0.006 | 1.87E-16 |  | 0.031 | 0.015 | 0.034 |
| rs34670419 | 7 | 99130834 | T | G | -0.088 | 0.015 | 1.09E-08 |  | 0.023 | 0.024 | 0.337 |
| rs35125553 | 12 | 1639249 | G | A | 0.038 | 0.007 | 5.20E-09 |  | -0.011 | 0.016 | 0.507 |
| rs35199438 | 11 | 16630779 | T | G | -0.049 | 0.006 | 2.36E-15 |  | -0.021 | 0.014 | 0.130 |
| rs3743347 | 15 | 67547301 | A | C | 0.052 | 0.007 | 1.75E-14 |  | -0.028 | 0.018 | 0.115 |
| rs3801387 | 7 | 120974765 | G | A | 0.135 | 0.006 | 1.15E-100 |  | 0.005 | 0.016 | 0.776 |
| rs4757350 | 11 | 15703674 | T | C | -0.056 | 0.007 | 3.75E-16 |  | -0.019 | 0.015 | 0.180 |
| rs4846580 | 1 | 219897941 | A | G | 0.035 | 0.006 | 3.21E-09 |  | 0.028 | 0.014 | 0.039 |
| rs55781332 | 11 | 242859 | G | A | 0.055 | 0.007 | 8.07E-16 |  | 0.002 | 0.015 | 0.915 |
| rs56104760 | 1 | 22486029 | G | A | -0.075 | 0.007 | 7.38E-24 |  | 0.008 | 0.016 | 0.617 |
| rs6029130 | 20 | 39103882 | T | C | 0.035 | 0.006 | 3.50E-08 |  | -0.016 | 0.017 | 0.352 |
| rs6040063 | 20 | 10640877 | G | A | -0.036 | 0.006 | 1.78E-10 |  | 0.001 | 0.014 | 0.969 |
| rs61884327 | 11 | 46766890 | C | T | 0.080 | 0.010 | 4.63E-16 |  | -0.073 | 0.029 | 0.011 |
| rs633995 | 1 | 172186729 | A | G | 0.035 | 0.006 | 1.61E-09 |  | 0.004 | 0.014 | 0.806 |
| rs634277 | 11 | 86887931 | G | A | -0.061 | 0.006 | 2.15E-23 |  | 0.034 | 0.014 | 0.019 |
| rs6465511 | 7 | 96134115 | G | C | 0.074 | 0.006 | 1.03E-34 |  | 0.012 | 0.015 | 0.419 |
| rs6557155 | 6 | 151910126 | G | T | 0.075 | 0.006 | 2.56E-37 |  | 0.004 | 0.014 | 0.761 |
| rs6960249 | 7 | 96660132 | G | T | -0.033 | 0.006 | 1.45E-08 |  | -0.041 | 0.014 | 0.003 |
| rs71390846 | 16 | 86714715 | C | G | -0.048 | 0.008 | 1.38E-10 |  | -0.006 | 0.017 | 0.735 |
| rs725670 | 11 | 121913230 | A | G | -0.032 | 0.006 | 3.61E-08 |  | -0.015 | 0.014 | 0.288 |
| rs73169678 | 7 | 150953205 | A | C | 0.062 | 0.009 | 1.05E-11 |  | -0.017 | 0.021 | 0.428 |
| rs73305797 | 7 | 30997087 | T | A | -0.042 | 0.007 | 2.40E-10 |  | 0.009 | 0.018 | 0.617 |
| rs73349318 | 10 | 112245400 | T | A | 0.047 | 0.009 | 2.68E-08 |  | -0.004 | 0.019 | 0.846 |
| rs73719807 | 7 | 121191251 | C | A | 0.093 | 0.011 | 1.14E-16 |  | 0.025 | 0.032 | 0.428 |
| rs74394007 | 3 | 156692207 | C | A | -0.061 | 0.008 | 2.46E-13 |  | -0.032 | 0.017 | 0.065 |
| rs7548588 | 1 | 110475971 | C | T | 0.037 | 0.006 | 2.21E-10 |  | -0.028 | 0.014 | 0.039 |
| rs757138 | 7 | 27989403 | G | T | 0.035 | 0.006 | 3.33E-08 |  | 0.013 | 0.015 | 0.398 |
| rs7586085 | 2 | 166577489 | G | A | -0.053 | 0.006 | 8.64E-21 |  | -0.019 | 0.014 | 0.157 |
| rs76051363 | 4 | 1006987 | T | C | -0.079 | 0.009 | 1.39E-20 |  | -0.027 | 0.019 | 0.147 |
| rs7728694 | 5 | 88288341 | T | G | -0.050 | 0.006 | 1.30E-17 |  | -0.003 | 0.014 | 0.840 |
| rs7740042 | 6 | 151971720 | A | T | -0.049 | 0.007 | 2.71E-12 |  | 0.021 | 0.020 | 0.272 |
| rs7741085 | 6 | 44636919 | T | C | 0.042 | 0.006 | 1.51E-13 |  | -0.003 | 0.014 | 0.812 |
| rs78667121 | 13 | 43200103 | A | G | 0.133 | 0.018 | 1.70E-13 |  | 0.035 | 0.032 | 0.281 |
| rs8047501 | 16 | 392318 | G | A | -0.052 | 0.006 | 1.13E-18 |  | -0.009 | 0.014 | 0.504 |
| rs8070128 | 17 | 17804725 | T | C | -0.039 | 0.006 | 1.98E-11 |  | 0.008 | 0.014 | 0.568 |
| rs818427 | 5 | 112221869 | T | C | 0.034 | 0.006 | 2.37E-08 |  | 0.019 | 0.014 | 0.186 |
| rs838721 | 2 | 234324232 | G | A | 0.031 | 0.006 | 4.48E-08 |  | -0.018 | 0.014 | 0.192 |
| rs884205 | 18 | 60054857 | C | A | 0.053 | 0.007 | 4.39E-15 |  | 0.013 | 0.015 | 0.401 |
| rs9594738 | 13 | 42952145 | T | C | -0.061 | 0.006 | 3.84E-27 |  | -0.011 | 0.014 | 0.423 |
| rs9910055 | 17 | 42283037 | T | C | 0.044 | 0.007 | 3.12E-11 |  | -0.004 | 0.015 | 0.804 |
| rs9972944 | 17 | 63771079 | G | A | -0.036 | 0.006 | 6.87E-10 |  | 0.043 | 0.014 | 0.002 |
| rs9976876 | 21 | 36970350 | T | G | -0.038 | 0.006 | 8.01E-11 |  | 0.021 | 0.014 | 0.130 |

EA, effect allele; OA, other allele; SNP, single nucleotide polymorphism; SE, standard error.
