# Supplementary material for: Dried fruit intake causally protects against low back pain: A Mendelian randomization study
Source: Front Nutr. 2023 Mar 23;10:1027481. doi: 10.3389/fnut.2023.1027481 (PMC10076586; doi:10.3389/fnut.2023.1027481)
Supplement: Supplementary file 9 [file Table_9.DOCX]

Supplementary Table S9 Characteristics of the instruments for serum 25-hydroxyvitamin D levels and their associations with low back pain.

| **SNP** | **Chr** | **Position** | **EA** | **OA** | **Exposure effect** |  |  |  | **Outcome effect** |  |  |
| --- | --- | --- | --- | --- | --- | --- | --- | --- | --- | --- | --- |
|  |  |  |  |  | **β** | **SE** | ***P*** |  | **β** | **SE** | ***P*** |
| rs10277163 | 7 | 21569089 | G | A | -0.014 | 0.002 | 1.08E-09 |  | 0.004 | 0.016 | 0.796 |
| rs1038165 | 12 | 68665940 | T | C | 0.012 | 0.002 | 2.15E-08 |  | 0.006 | 0.014 | 0.678 |
| rs1042034 | 2 | 21225281 | T | C | -0.015 | 0.003 | 1.45E-09 |  | -0.016 | 0.015 | 0.282 |
| rs10438978 | 18 | 47158186 | C | T | -0.017 | 0.003 | 7.34E-11 |  | 0.008 | 0.018 | 0.670 |
| rs1047891 | 2 | 211540507 | A | C | -0.013 | 0.002 | 7.96E-10 |  | 0.016 | 0.015 | 0.284 |
| rs1048328 | 19 | 51527364 | A | G | 0.031 | 0.004 | 5.58E-17 |  | 0.002 | 0.023 | 0.926 |
| rs10859995 | 12 | 96375682 | C | T | -0.044 | 0.002 | 4.60E-100 |  | 0.005 | 0.014 | 0.731 |
| rs11023159 | 11 | 14262063 | C | T | 0.048 | 0.006 | 3.73E-17 |  | -0.067 | 0.040 | 0.095 |
| rs11076175 | 16 | 57006378 | G | A | 0.023 | 0.003 | 9.64E-18 |  | 0.032 | 0.018 | 0.076 |
| rs111515741 | 11 | 14370944 | A | G | -0.049 | 0.008 | 3.95E-10 |  | 0.200 | 0.112 | 0.074 |
| rs11207969 | 1 | 62911751 | G | A | 0.021 | 0.002 | 7.14E-23 |  | 0.023 | 0.015 | 0.133 |
| rs11264361 | 1 | 155289545 | G | T | 0.017 | 0.002 | 7.97E-14 |  | 0.021 | 0.015 | 0.157 |
| rs1128535 | 3 | 49866392 | T | C | 0.016 | 0.002 | 6.06E-16 |  | 0.049 | 0.014 | 0.001 |
| rs115288876 | 1 | 152000117 | A | G | 0.079 | 0.005 | 2.36E-56 |  | 0.038 | 0.035 | 0.268 |
| rs11542462 | 16 | 82033810 | A | G | -0.025 | 0.003 | 9.72E-17 |  | 0.011 | 0.023 | 0.641 |
| rs11600054 | 11 | 14690511 | A | G | 0.068 | 0.010 | 1.84E-11 |  | 0.058 | 0.048 | 0.227 |
| rs11726886 | 4 | 72822599 | A | C | -0.054 | 0.002 | 3.08E-125 |  | -0.019 | 0.015 | 0.184 |
| rs117300835 | 11 | 15118975 | A | G | -0.335 | 0.009 | 1.00E-200 |  | 0.066 | 0.063 | 0.296 |
| rs11791258 | 9 | 107632644 | A | G | 0.014 | 0.003 | 4.85E-08 |  | -0.002 | 0.021 | 0.943 |
| rs11867297 | 17 | 66433493 | T | C | 0.014 | 0.002 | 1.01E-10 |  | 0.008 | 0.014 | 0.597 |
| rs12056768 | 8 | 116988527 | G | T | -0.023 | 0.002 | 2.65E-29 |  | 0.013 | 0.014 | 0.334 |
| rs12153819 | 6 | 83824703 | T | C | -0.018 | 0.003 | 8.16E-09 |  | 0.019 | 0.018 | 0.308 |
| rs12283049 | 11 | 14690192 | G | A | -0.056 | 0.002 | 9.62E-122 |  | 0.009 | 0.017 | 0.620 |
| rs12324720 | 15 | 64092140 | A | G | -0.015 | 0.003 | 2.45E-08 |  | 0.019 | 0.016 | 0.244 |
| rs12462826 | 19 | 11955767 | A | G | -0.013 | 0.002 | 4.18E-10 |  | -0.003 | 0.014 | 0.854 |
| rs12501515 | 4 | 72592838 | A | G | -0.079 | 0.002 | 1.00E-200 |  | -0.026 | 0.014 | 0.061 |
| rs1260326 | 2 | 27730940 | C | T | 0.020 | 0.002 | 1.96E-21 |  | -0.013 | 0.014 | 0.350 |
| rs12775091 | 10 | 91524012 | T | C | 0.016 | 0.002 | 3.33E-10 |  | -0.014 | 0.015 | 0.353 |
| rs13076508 | 3 | 52407805 | C | T | 0.025 | 0.005 | 2.78E-08 |  | -0.003 | 0.034 | 0.933 |
| rs13108245 | 4 | 57790205 | G | A | -0.012 | 0.002 | 4.63E-09 |  | -0.004 | 0.014 | 0.754 |
| rs1321247 | 6 | 25662873 | T | A | -0.022 | 0.003 | 4.36E-11 |  | -0.026 | 0.021 | 0.229 |
| rs13294734 | 9 | 80710910 | T | C | 0.013 | 0.002 | 1.02E-09 |  | 0.012 | 0.014 | 0.387 |
| rs1343776 | 1 | 41757718 | A | G | 0.018 | 0.002 | 1.62E-13 |  | 0.030 | 0.016 | 0.066 |
| rs138335 | 22 | 41227086 | G | C | -0.014 | 0.002 | 1.56E-10 |  | -0.013 | 0.014 | 0.371 |
| rs1384687 | 8 | 61525963 | A | G | -0.017 | 0.003 | 1.82E-08 |  | -0.018 | 0.015 | 0.244 |
| rs142004400 | 14 | 50829560 | C | A | -0.031 | 0.006 | 3.01E-08 |  | -0.053 | 0.048 | 0.269 |
| rs142158911 | 19 | 11190534 | A | G | 0.026 | 0.003 | 4.43E-16 |  | 0.008 | 0.022 | 0.708 |
| rs144965707 | 11 | 14093639 | A | G | -0.035 | 0.004 | 1.52E-16 |  | -0.042 | 0.030 | 0.164 |
| rs1532085 | 15 | 58683366 | G | A | 0.025 | 0.002 | 8.60E-34 |  | 0.016 | 0.014 | 0.236 |
| rs1627043 | 11 | 71110175 | C | G | -0.049 | 0.006 | 8.49E-18 |  | 0.047 | 0.036 | 0.182 |
| rs1684600 | 16 | 4594671 | T | C | -0.013 | 0.002 | 1.59E-08 |  | -0.008 | 0.016 | 0.604 |
| rs17207784 | 6 | 22768668 | C | T | -0.013 | 0.002 | 5.14E-10 |  | 0.012 | 0.015 | 0.430 |
| rs17473257 | 11 | 14283186 | A | G | -0.061 | 0.008 | 4.59E-15 |  | 0.038 | 0.053 | 0.471 |
| rs1800588 | 15 | 58723675 | T | C | -0.031 | 0.002 | 4.73E-35 |  | 0.046 | 0.016 | 0.004 |
| rs1841850 | 20 | 52718555 | C | A | 0.030 | 0.003 | 6.73E-22 |  | 0.012 | 0.017 | 0.511 |
| rs1858889 | 7 | 107117447 | C | A | 0.013 | 0.002 | 3.49E-11 |  | 0.001 | 0.014 | 0.950 |
| rs1871395 | 12 | 21352315 | G | A | -0.020 | 0.003 | 5.72E-13 |  | 0.022 | 0.015 | 0.140 |
| rs1949633 | 3 | 153758806 | C | T | 0.011 | 0.002 | 4.45E-08 |  | 0.010 | 0.014 | 0.475 |
| rs2037511 | 18 | 61366207 | A | G | 0.018 | 0.003 | 9.41E-11 |  | -0.004 | 0.018 | 0.839 |
| rs2074735 | 22 | 31535872 | C | G | 0.029 | 0.004 | 1.22E-12 |  | 0.022 | 0.019 | 0.260 |
| rs2171427 | 12 | 24822366 | A | G | -0.017 | 0.003 | 4.26E-09 |  | 0.008 | 0.019 | 0.688 |
| rs2229742 | 21 | 16339172 | C | G | -0.025 | 0.003 | 4.75E-14 |  | -0.031 | 0.022 | 0.149 |
| rs2245133 | 6 | 131931092 | C | T | -0.021 | 0.003 | 7.80E-15 |  | -0.006 | 0.016 | 0.701 |
| rs2297991 | 10 | 113913222 | C | T | 0.013 | 0.002 | 1.57E-08 |  | -0.002 | 0.014 | 0.918 |
| rs2398113 | 10 | 10078742 | G | A | -0.012 | 0.002 | 1.10E-08 |  | -0.008 | 0.014 | 0.557 |
| rs2494429 | 1 | 2339395 | G | A | -0.015 | 0.003 | 2.80E-08 |  | 0.016 | 0.018 | 0.365 |
| rs2511279 | 11 | 71130419 | G | C | 0.098 | 0.005 | 2.98E-79 |  | 0.006 | 0.027 | 0.820 |
| rs2595644 | 7 | 43980540 | T | G | -0.012 | 0.002 | 4.97E-09 |  | -0.008 | 0.014 | 0.585 |
| rs2710651 | 2 | 63166379 | A | G | -0.012 | 0.002 | 1.23E-08 |  | 0.002 | 0.014 | 0.890 |
| rs2756119 | 14 | 104001517 | A | G | 0.012 | 0.002 | 8.71E-09 |  | 0.012 | 0.014 | 0.410 |
| rs2807834 | 1 | 220970593 | G | T | -0.015 | 0.002 | 5.66E-12 |  | -0.003 | 0.015 | 0.818 |
| rs28435470 | 12 | 133067473 | A | G | -0.012 | 0.002 | 3.29E-08 |  | 0.008 | 0.014 | 0.561 |
| rs2847500 | 11 | 120114421 | A | G | -0.023 | 0.003 | 2.77E-13 |  | -0.009 | 0.019 | 0.625 |
| rs290400 | 20 | 52698179 | A | G | -0.013 | 0.002 | 1.41E-09 |  | 0.021 | 0.014 | 0.133 |
| rs3114045 | 4 | 100252560 | C | T | -0.022 | 0.003 | 1.00E-13 |  | -0.005 | 0.020 | 0.822 |
| rs325393 | 15 | 100229260 | T | G | -0.014 | 0.002 | 2.03E-09 |  | -0.027 | 0.015 | 0.058 |
| rs34186890 | 3 | 141720712 | G | A | -0.016 | 0.002 | 1.33E-11 |  | 0.002 | 0.016 | 0.920 |
| rs34726834 | 8 | 25889606 | T | C | 0.014 | 0.002 | 2.42E-09 |  | 0.004 | 0.015 | 0.813 |
| rs35270497 | 2 | 38259872 | T | C | 0.016 | 0.003 | 5.08E-09 |  | -0.004 | 0.017 | 0.815 |
| rs35823191 | 1 | 17560123 | C | T | -0.023 | 0.002 | 1.65E-27 |  | 0.020 | 0.014 | 0.165 |
| rs3732220 | 2 | 234627048 | A | G | -0.048 | 0.004 | 1.31E-39 |  | -0.017 | 0.026 | 0.523 |
| rs3829251 | 11 | 71194559 | A | G | -0.114 | 0.003 | 1.00E-200 |  | 0.010 | 0.015 | 0.520 |
| rs4147536 | 4 | 100239112 | C | A | -0.015 | 0.002 | 2.76E-09 |  | -0.025 | 0.016 | 0.114 |
| rs4348160 | 4 | 70017531 | G | T | -0.026 | 0.002 | 6.62E-33 |  | 0.005 | 0.014 | 0.697 |
| rs4364259 | 4 | 15892159 | A | G | 0.017 | 0.003 | 1.86E-11 |  | -0.003 | 0.016 | 0.867 |
| rs4420638 | 19 | 45422946 | G | A | -0.019 | 0.003 | 3.95E-13 |  | -0.027 | 0.015 | 0.082 |
| rs4580037 | 13 | 55702646 | C | A | -0.014 | 0.002 | 1.68E-09 |  | -0.020 | 0.015 | 0.180 |
| rs512083 | 1 | 46027355 | C | T | 0.012 | 0.002 | 2.23E-09 |  | 0.001 | 0.014 | 0.925 |
| rs5770794 | 22 | 50880781 | T | C | -0.013 | 0.002 | 1.74E-09 |  | 0.020 | 0.014 | 0.146 |
| rs6129648 | 20 | 39231118 | G | A | 0.014 | 0.002 | 2.44E-11 |  | 0.016 | 0.014 | 0.271 |
| rs61698755 | 17 | 79257880 | C | T | -0.011 | 0.002 | 2.25E-08 |  | -0.021 | 0.014 | 0.130 |
| rs61747728 | 1 | 179526214 | T | C | 0.030 | 0.005 | 8.83E-09 |  | 0.029 | 0.028 | 0.290 |
| rs61813875 | 1 | 152536650 | G | C | 0.082 | 0.007 | 1.16E-35 |  | 0.046 | 0.077 | 0.545 |
| rs61887421 | 11 | 70949673 | C | T | -0.037 | 0.006 | 8.05E-10 |  | 0.008 | 0.039 | 0.847 |
| rs62007299 | 15 | 77711719 | A | G | -0.012 | 0.002 | 3.12E-08 |  | -0.001 | 0.014 | 0.959 |
| rs635634 | 9 | 136155000 | T | C | -0.015 | 0.003 | 7.55E-09 |  | -0.003 | 0.017 | 0.848 |
| rs6438900 | 3 | 125148287 | G | C | 0.015 | 0.002 | 1.27E-10 |  | 0.010 | 0.015 | 0.507 |
| rs6834488 | 4 | 88178919 | T | C | -0.014 | 0.002 | 2.26E-12 |  | 0.011 | 0.015 | 0.430 |
| rs71599974 | 4 | 71765339 | G | A | 0.026 | 0.003 | 2.39E-19 |  | -0.001 | 0.020 | 0.957 |
| rs727857 | 2 | 58981967 | A | G | -0.012 | 0.002 | 9.27E-09 |  | -0.005 | 0.014 | 0.736 |
| rs733454 | 11 | 76477721 | T | C | 0.019 | 0.003 | 2.93E-08 |  | 0.038 | 0.030 | 0.206 |
| rs73413596 | 12 | 111582630 | C | T | 0.022 | 0.004 | 9.15E-09 |  | 0.012 | 0.029 | 0.672 |
| rs742493 | 6 | 40998167 | C | T | 0.018 | 0.003 | 1.04E-08 |  | 0.048 | 0.023 | 0.037 |
| rs7528419 | 1 | 109817192 | G | A | 0.022 | 0.002 | 8.17E-19 |  | 0.000 | 0.017 | 0.994 |
| rs7569755 | 2 | 118648261 | A | G | 0.014 | 0.002 | 1.49E-09 |  | 0.028 | 0.017 | 0.090 |
| rs7580771 | 2 | 101428119 | T | G | -0.017 | 0.003 | 5.15E-10 |  | -0.021 | 0.016 | 0.187 |
| rs7652808 | 3 | 85603643 | G | T | -0.021 | 0.002 | 1.36E-23 |  | 0.000 | 0.015 | 0.977 |
| rs7712001 | 5 | 148020950 | G | T | 0.012 | 0.002 | 7.05E-09 |  | -0.014 | 0.014 | 0.322 |
| rs77532868 | 10 | 88081438 | T | C | 0.026 | 0.005 | 1.28E-08 |  | 0.008 | 0.039 | 0.831 |
| rs7784802 | 7 | 64017227 | T | A | 0.013 | 0.002 | 3.06E-10 |  | -0.022 | 0.015 | 0.137 |
| rs77924615 | 16 | 20392332 | A | G | -0.015 | 0.003 | 3.94E-09 |  | 0.005 | 0.016 | 0.747 |
| rs77960347 | 18 | 47109955 | G | A | -0.053 | 0.009 | 6.53E-09 |  | -0.029 | 0.080 | 0.721 |
| rs78649910 | 4 | 3482213 | A | T | -0.019 | 0.003 | 8.33E-09 |  | -0.004 | 0.021 | 0.847 |
| rs8018720 | 14 | 39556185 | C | G | -0.034 | 0.003 | 1.94E-38 |  | 0.013 | 0.019 | 0.491 |
| rs804281 | 8 | 11611865 | G | A | 0.016 | 0.002 | 1.20E-14 |  | -0.020 | 0.019 | 0.312 |
| rs8107974 | 19 | 19388500 | T | A | 0.036 | 0.004 | 1.36E-20 |  | -0.013 | 0.028 | 0.638 |
| rs8121940 | 20 | 52742306 | G | C | -0.044 | 0.003 | 1.77E-65 |  | 0.029 | 0.016 | 0.073 |
| rs9375037 | 6 | 121856794 | C | A | 0.012 | 0.002 | 1.21E-08 |  | -0.008 | 0.014 | 0.582 |
| rs9409266 | 9 | 125745042 | A | G | -0.017 | 0.003 | 1.24E-08 |  | 0.007 | 0.018 | 0.695 |
| rs964184 | 11 | 116648917 | C | G | 0.041 | 0.003 | 3.50E-42 |  | -0.004 | 0.019 | 0.824 |
| rs9847248 | 3 | 18804655 | A | G | -0.012 | 0.002 | 4.19E-08 |  | 0.019 | 0.014 | 0.194 |
| rs986649 | 5 | 118668050 | G | A | 0.013 | 0.002 | 3.51E-09 |  | -0.015 | 0.014 | 0.298 |
| rs9946771 | 18 | 28918628 | T | C | -0.023 | 0.004 | 9.47E-09 |  | 0.016 | 0.025 | 0.527 |

EA, effect allele; OA, other allele; SNP, single nucleotide polymorphism; SE, standard error.
