# Supplementary material for: Dried fruit intake causally protects against low back pain: A Mendelian randomization study
Source: Front Nutr. 2023 Mar 23;10:1027481. doi: 10.3389/fnut.2023.1027481 (PMC10076586; doi:10.3389/fnut.2023.1027481)
Supplement: Supplementary file 10 [file Table_10.DOCX]

Supplementary Table S10 Characteristics of the instruments for vigorous physical activity and their associations with low back pain.

| **SNP** | **Chr** | **Position** | **EA** | **OA** | **Exposure effect** |  |  |  | **Outcome effect** |  |  |
| --- | --- | --- | --- | --- | --- | --- | --- | --- | --- | --- | --- |
|  |  |  |  |  | **β** | **SE** | ***P*** |  | **β** | **SE** | ***P*** |
| rs1248860 | 3 | 85015779 | A | G | 0.010 | 0.001 | 1.10E-13 |  | 0.012 | 0.014 | 0.396 |
| rs13243553 | 7 | 133506955 | A | G | -0.009 | 0.001 | 9.00E-11 |  | -0.008 | 0.014 | 0.594 |
| rs2764261 | 6 | 108927842 | G | A | -0.009 | 0.001 | 2.00E-11 |  | -0.005 | 0.014 | 0.692 |
| rs328902 | 7 | 35020843 | T | C | 0.009 | 0.001 | 5.50E-10 |  | -0.012 | 0.015 | 0.424 |
| rs3781411 | 10 | 126715436 | T | C | -0.013 | 0.002 | 3.00E-10 |  | 0.029 | 0.018 | 0.107 |
| rs6667222 | 1 | 154253661 | C | A | -0.009 | 0.002 | 8.70E-09 |  | 0.013 | 0.016 | 0.414 |
| rs9276758 | 6 | 32772975 | A | G | -0.008 | 0.001 | 1.40E-08 |  | 0.026 | 0.015 | 0.079 |

EA, effect allele; OA, other allele; SNP, single nucleotide polymorphism; SE, standard error.
